# Higher eigenvector centrality in grooming network is linked to better inhibitory control task performance but not other cognitive tasks in free-ranging Japanese macaques

**DOI:** 10.1038/s41598-024-77912-7

**Published:** 2024-11-19

**Authors:** Yu Kaigaishi, Shinya Yamamoto

**Affiliations:** 1 Institute for Advanced Study, Kyoto University, Kyoto, Japan; 2 Wildlife Research Center, Kyoto University, Kyoto, Japan

**Keywords:** Social Intelligence Hypothesis, Social network analysis, Field experiment, Cognitive test battery, Japanese macaque, Animal behaviour, Behavioural ecology, Psychology

## Abstract

The Social Intelligence Hypothesis predicts that complex social environments promote higher cognitive capacities. This hypothesis is often tested by comparing species or conspecific groups with varying group sizes or social structures. However, individual social environments differ not only among species or groups but also within the same group. We examined the relationship between social centrality and cognitive ability in wild Japanese macaques (*Macaca fuscata*). We created a grooming network using data from 196 adult macaques and calculated social centrality for each individual. We then tested the macaques with a cognitive test battery to assess their abilities in social, physical, and inhibitory control domains. Our findings revealed that social centrality was uniquely associated with inhibitory control performance, particularly in the behavioral inhibition task, but not with other cognitive domains. This suggests that inhibitory control, a key component of executive functions, plays a pivotal role in the social lives of wild Japanese macaques.

**Supplementary Information:**

The online version contains supplementary material available at 10.1038/s41598-024-77912-7.

## Introduction

Most primate species, including humans, reside in stable social groups consisting of multiple individuals. Individuals in a group interact with other individuals in various ways and contexts, resulting in the development of diverse relationships through the accumulation of social interactions^1^. Such relationships lead to higher stability and cohesiveness of the whole group structure, which increases individual fitness through various benefits, such as predator avoidance and efficient foraging^2,3^. Research has demonstrated that strong and stable social bonds confer numerous benefits to individuals, such as higher fitness, increased longevity, mitigated stress levels and improved health^4,5^. However, maintaining social ties may also impose high cognitive costs on individuals^6,7^, such as coordinating multiple relationships and recognizing and tracking third-party relationships to make various social decisions^7,8^. It has been hypothesized that such cognitive demands limit the size and properties of an individual’s or species’ social network^9,10^.

The Social Intelligence Hypothesis (SIH) proposes that cognitive challenges accompanied by social living enhance the evolution of advanced cognitive abilities in animal species^11–13^. Most empirical research testing this hypothesis has involved inter-species comparisons, with the prediction that species with more complex societies show more sophisticated cognition. There is abundant evidence supporting this hypothesis, such as the positive association between species’ typical group sizes and brain structures^9,13^ or socio-cognitive abilities^14^. Interestingly, similar findings have been observed from intraspecific studies that investigated the relationship between group size and cognitive performance. For instance, larger group sizes were found to be associated with better cognitive abilities in wild magpies (*Gymnorhina tibicen*)^15^ and hyenas (*Crocuta crocuta*)^16^, as well as larger brain regions involved in social cognition in captive rhesus macaques (*Macaca mulatta*)^17,18^. These studies suggest that complex social environments may lead to the evolution of more sophisticated cognition across various animal taxa, and even within the same species. Nevertheless, it is important to acknowledge that several studies have presented evidence against the association between sociality and cognition^19,20^, highlighting ongoing debates about what factors are involved in the evolution of cognitive abilities^21,22^.

An unexplored question about the SIH is whether and how individual variations in sociality are related to cognitive performance in non-human animals^23^. Even within the same group, individuals experience different social environments depending on the number of social partners, the frequency of interaction with them, and the properties of the partners’ social network (i.e., friends of friends)^24,25^. If, as predicted by the SIH^6,7^, social lives impose cognitive demands on animals, then differences in sociality at the individual level would also affect cognitive abilities. Notably, researchers have found that such individual variations in sociality are associated with cognitive abilities in humans. Several studies revealed that social network sizes are positively correlated with brain regional volumes associated with social cognition or socio-cognitive task performance, such as perspective-taking and social working memory^26–29^. Moreover, a meta-analysis revealed that involvement in larger social networks improved general cognitive abilities among older adults^30^. These studies indicate that daily social life affects one’s cognitive abilities among humans.

In contrast, only a few studies have examined the relationship between cognition and sociality at the individual level among non-human animals. In carrion crows (*Corvus corone*), the frequency of social interactions was correlated with cognitive task performance, such as the delay of gratification and inequity aversion^31^. Among primate species, Testard et al.^32^ observed rhesus macaques and found that having more grooming partners was associated with larger volumes in the mid-superior temporal sulcus and ventral dysgranular insula, which are brain regions activated specifically during social contexts^33,34^. While this study suggested a link between individual sociality and specific brain regions, they did not directly examine what cognitive traits were involved. Given that brain anatomical variations may not fully reflect cognitive performance^35^, a more direct investigation into cognitive performance and individual sociality is necessary to test the SIH at an individual level.

In this study, we explored the relationship between sociality and several domains of cognition at the individual level with free-ranging Japanese macaques (*Macaca fuscata*). To this end, we integrated social network analyses with cognitive experiments in the field. Specifically, we measured several social centrality indices in the grooming network as individual sociality. Social centrality measures the role and position each individual occupies in a network by evaluating how well an individual is connected with other members in a network from various perspectives. The simplest measures include degree (the number of social partners) and strength (the frequency of social interactions an individual has). Furthermore, social centralities can be calculated using indirect connections, such as the number of partners one’s partners have (eigenvector centrality), the significance of an individual in bridging the connections of the whole network (betweenness centrality), or how easily an individual can reach all others in the network (closeness centrality). To ascertain what cognitive areas are particularly associated with sociality among Japanese macaques, we developed a cognitive test battery with multiple cognitive tasks that consist of social cognition, physical cognition, and inhibitory control ability tasks.

We predicted that performance in social cognitive tasks would be associated with social centralities if the SIH is applicable at an individual level within species. More specifically, we predicted a positive association between the number of grooming partners and socio-cognitive task performance given the recent findings in rhesus macaques^32^. For further investigation, we adopted two social cognition tasks, which were designed to simulate socially cooperative (social cue task) or competitive situations (gaze-perception task). With these two types of social cognition tasks, we investigated whether competitive or cooperative skills are more closely linked to the individual’s social centrality in a group. We did not make specific predictions about which task would be associated with any types of social centralities. While the tasks of the physical domain covered the three scales of physical cognition that was proposed in previous studies (space: spatial memory, transposition; quantities: relative number; causality: noise)^36–39^, we predicted that none of the tasks of this domain would be linked to social centralities.

Furthermore, we predicted that inhibitory control ability, a key concept of executive functions essential for behavioral flexibility^40^, would be positively correlated with some type of social centralities. Evidence from various animal taxa suggests that higher levels of inhibitory control are related to higher social complexity^15,16,41^ (but negative associations between sociality and inhibition were observed in lemurs^14^ and guppies^42^). For instance, in primates, higher levels of inhibitory control were associated with a higher degree of fission-fusion dynamics^41^. This may reflect the need for increased behavioral flexibility for living in a society where ranging group membership regularly changes^41^. While these studies focused on inter-specific variations in sociality, the ability to make adaptable social decisions would be crucial even within a single group. Thus, at the individual level, higher social centrality may be indicative of superior inhibitory control abilities. For the inhibitory domain, we employed two widely recognized tasks, the cylinder task and the A-not-B task. We selected these two tasks because they are considered to measure different components of inhibition, namely behavioral and cognitive inhibition^40,43^. Thus, by employing these two, we aimed to test which aspect of inhibitory control would be linked to any types of social centralities.

## Results

### Social network traits

**Fig. 1:**
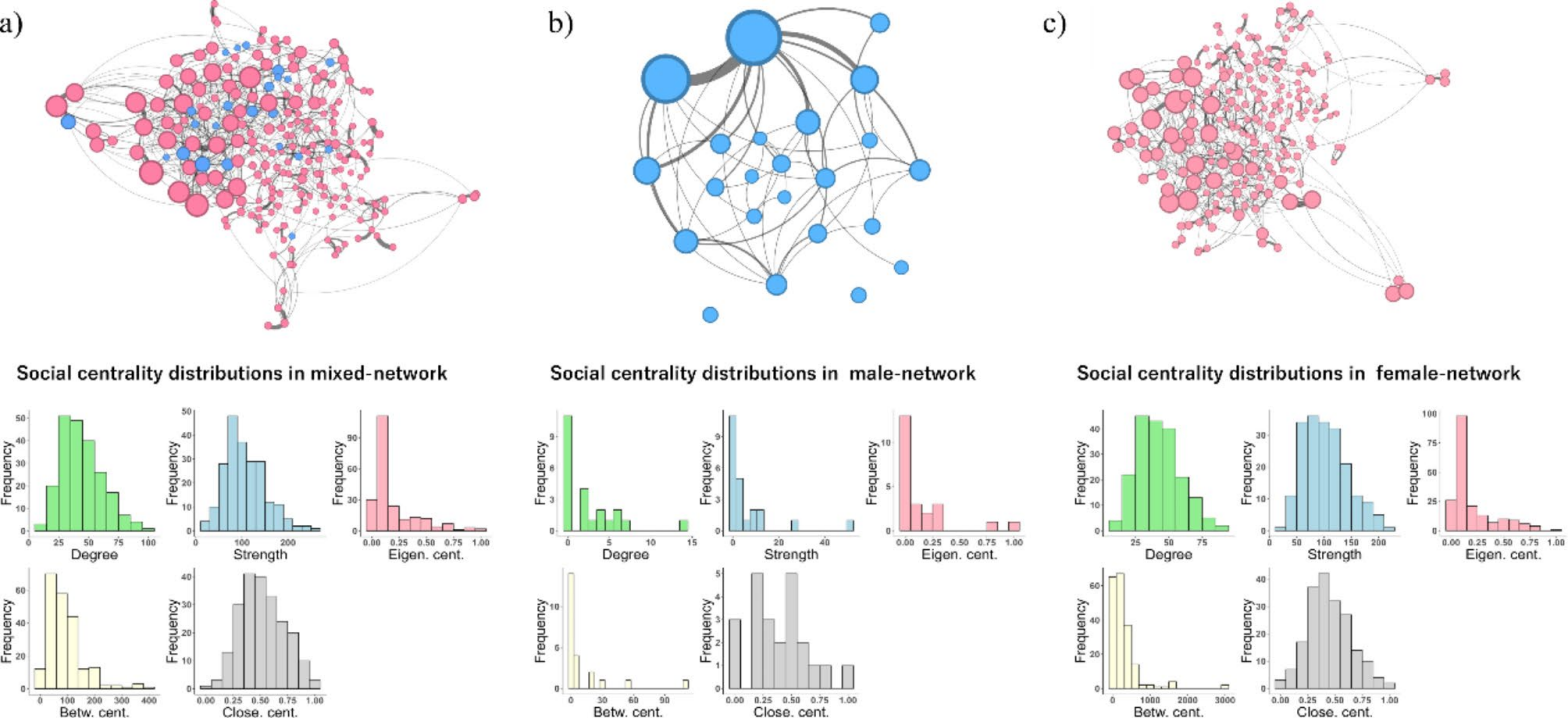
Grooming networks (upper) and histograms showing the distribution of social centralities in each network (lower). Each node represents an individual macaque and the edges between the nodes represent the existence of grooming interactions between them. The size of the nodes reflects the value of eigenvector centrality of each individual. Thickness of the lines reflect the frequency of grooming. Blue nodes indicate males and pink nodes indicate females. (**a**): Grooming network containing all individuals (*n* = 219); (**b**): Grooming network containing only males (*n* = 24); (**c**): Grooming network containing only females (*n* = 172). Note that for the networks (a) and (c), we depicted the edges with the weight smaller than the average value (a: 2.91, c: 2.97), to enhance the readability of the figure. We used the original network with no edge reduction for the analyzes.

We built three types of social networks based on grooming interactions among adult Japanese macaques from three years of observation. We calculated five social centrality measures: degree (the number of social partners), strength (the frequency of social interactions), eigenvector centrality (a measure of social integration considering direct and indirect social connections), betweenness centrality (the extent to which an individual bridges the entire social network), and closeness centrality (a measure of distance between an individual to all others in the network).The mixed-sex grooming network contained 218 individuals (23 males; 195 females). We found large variations in each centrality measure (degree: mean ± SD: 45.24 ± 17.09; strength: mean ± SD: 107.81 ± 44.35; eigenvector: mean ± SD: 0.19 ± 0.21; betweenness: mean ± SD: 92.47 ± 67.18; closeness: mean ± SD: 0.52 ± 0.20), suggesting the presence of significant inter-individual variation in sociality. The female grooming network, which included only female-female grooming, displayed topological characteristics like those of the mixed-sex network (degree: mean ± SD: 43.00 ± 15.73; strength: mean ± SD: 103.64 ± 40.26; eigenvector: mean ± SD: 0.20 ± 0.21; betweenness: mean ± SD: 286.60 ± 402.39; closeness: mean ± SD: 0.45 ± 0.20). In contrast, the male-network, based solely on male-male grooming interactions, had a different structure compared to the others, being highly centralized around two individuals. This resulted in the distributions of centralities being heavily skewed towards them (degree: mean ± SD: 2.48 ± 3.42; strength: mean ± SD: 5.87 ± 11.59; eigenvector: mean ± SD: 0.14 ± 0.27; betweenness: mean ± SD: 11.9 ± 25.25; closeness: mean ± SD: 0.39 ± 0.26). Owing to this topological characteristic, the social centralities within the male-grooming network, except for closeness, showed a high degree of correlation with each other (see Fig. S1b). The social networks and histograms of social centralities in each network are depicted in Fig. 1.

### Physical domain tasks

We conducted field experiments using a cognitive test battery to quantify the cognitive performance of each macaque, including physical (four tasks), social (two tasks) and inhibitory domains (two tasks). Regarding physical cognition, 80 subjects (9 males; 71 females) participated in the spatial memory and transposition tasks, 78 (9 males; 69 females) in the relative number task, and 75 (9 males; 66 females) in the noise task (Table 1). The macaques performed above chance level in the spatial memory (chance level = 0.33, mean success rate ± SD = 0.44 ± 0.19, *z* = 5.51, *p* < 0.001), transposition (chance level = 0.33, mean success rate ± SD = 0.37 ± 0.15, *z* = 4.08, *p* < 0.001) and relative number (chance level = 0.5, mean success rate ± SD = 0.63 ± 0.12, *z* = 6.03, *p* < 0.001), but not in the noise task (chance level = 0.5, mean success rate ± SD = 0.50 ± 0.11, *z* = 0.15, *p* = 0.89).

At the domain level, the full model explained the performance of the macaques better than the null model (LRT: χ^2^ = 118.39, *df* = 12, *p* < 0.01). However, only task type, and none of the social centrality measures, was significantly associated with physical domain task performance (see Table S1). Similar results were observed in the male- and female-network models, where only task type explained cognitive performances (male: χ^2^ = 31.80, *df* = 8, *p* < 0.01; female: χ^2^ = 94.93, *df* = 11, *p* < 0.01; see Table S1 and Supplementary Material S1). When analyzing each task separately, the full models did not explain the task performance better than the null models. Therefore, we found no effects of social centralities in the physical domain (see Supplementary Material S1 and Table S1 and 2 for the detailed GLMM results).

### Social domain tasks

Social domain consisted of two tasks, namely the social cue and the gaze perception task. Seventy-seven subjects (9 males; 68 females) participated in the social cue task and 89 (12 males; 77 females) in the gaze perception task (Table 1). In this domain, the macaques chose the correct option significantly above chance level both in the social cue (chance level = 0.5, mean success rate ± SD = 0.56 ± 0.13, *z* = 3.43, *p* < 0.001) and the gaze perception task (chance level = 0.5, mean success rate ± SD = 0.56 ± 0.18, *z* = 3.12, *p* = 0.002).

Domain-level analysis revealed that the full model did not explain trial success better than the null model in the mixed-sex (χ^2^ = 8.07, *df* = 10, *p* = 0.62), male-network (χ^2^ = 6.29, *df* = 7, *p* = 0.51) or female-network (χ^2^ = 9.04, *df* = 9, *p* = 0.44). Similarly, task-level analyses showed that the full models did not outperform the null models both for the social cue (mixed-sex: χ^2^ = 10.67, *df* = 10, *p* = 0.38; male: χ^2^ = 5.82, *df* = 6, *p* = 0.44; female: χ^2^ = 10.44, *df* = 9, *p* = 0.32) and the gaze perception task (mixed-sex: χ^2^ = 6.00, *df* = 10, *p* = 0.82; male: χ^2^ = 2.35, *df* = 7, *p* = 0.94; female: χ^2^ = 5.86, *df* = 9, *p* = 0.75). These results suggest that none of the explanatory variables including the social centralities were associated with the task performances in this domain (see Table S3 and 4 for the detailed GLMM results).

###  Inhibitory domain tasks

Eighty-six subjects (12 males; 74 females) participated in the A-not-B task and 119 (15 males; 105 females) in the cylinder task. The performance of the macaques in the A-not-B task did not differ significantly from chance level, although there was large inter-individual variation (chance level = 0.33, mean success rate ± SD = 0.37 ± 0.33, *z* = 0.18, *p* = 0.86). They also exhibited variable performances in the cylinder task, with the mean success rate of 0.54 and SD ± 0.30. Thus, when compared to the tasks of other domains, the performance of the macaques varied among individuals in the two inhibitory tasks (Table 1).

**Fig. 2:**
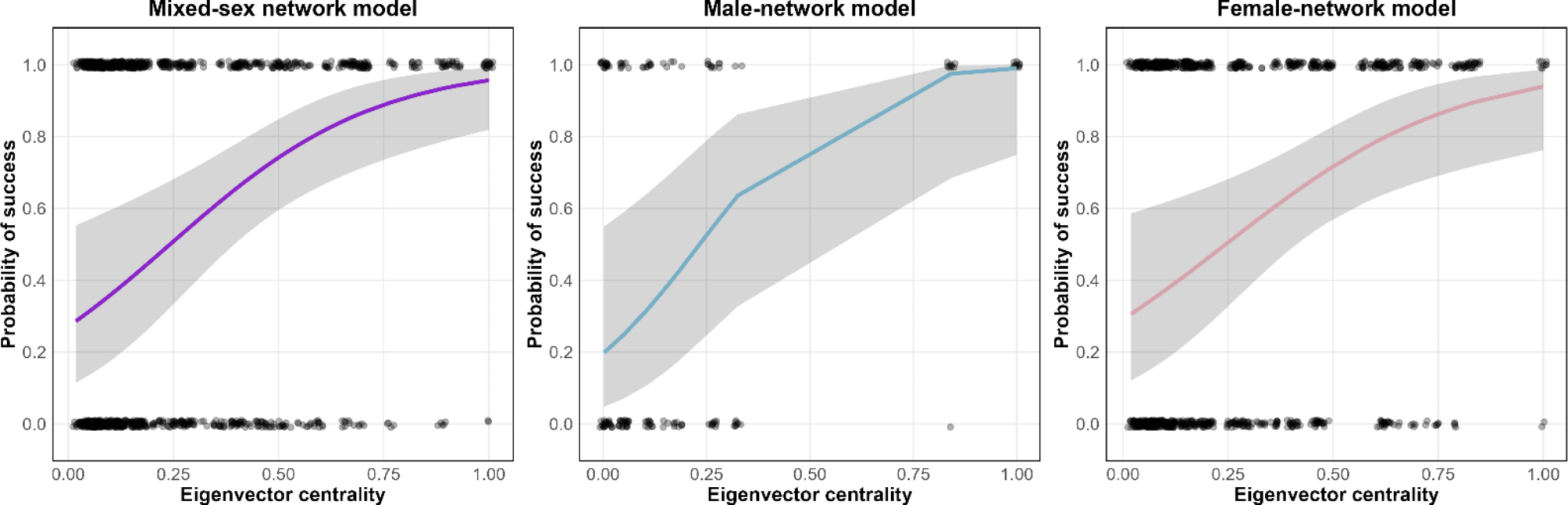
Predicted probability of success on the cylinder task in relation to the value of eigenvector centrality. Left: mixed-sex-network model; middle: male-network model; right; female-network model.

At the domain level, the full model provided a better explanation for the success of trials compared to the null model for mixed-sex (χ^2^ = 80.24, *df* = 10, *p* < 0.01), male-network (χ^2^ = 11.79, *df* = 5, *p* = 0.03) and female-network (χ^2^ = 77.84, *df* = 9, *p* < 0.01). Notably, we found significant and positive effects of eigenvector centrality, but not other centrality measures, in all three models (Fig. 2). This suggests that inhibitory control ability was particularly associated with the value of eigenvector centrality. Analyzing each task separately, we found that the effect of eigenvector centrality was significant only for the cylinder task but not the A-not-B task across all three models. Thus, higher eigenvector centrality was predictive of enhanced performance uniquely on the cylinder task, a task requiring inhibition of impulsiveness^20^. Other than social centrality measures, we also found that the macaques, especially females, performed better on the cylinder task than the A-not-B task. Additionally, there were significant and positive effects of trial number in both tasks, suggesting the presence of some learning effects. Finally, individuals belonging to the oldest age category showed worse performance in the A-not-B task (for the detailed GLMM results, see Supplementary Material S1 and Tables S5 and S6).

**Table 1:** Mean proportion of correct responses in each task of the cognitive test battery.

Task	Trials	Number of subjects	Chance level	Mean success rate ± SD
Physical				
Spatial memory	6	80	0.33	0.44 ± 0.19
Transposition	6	80	0.33	0.37 ± 0.15
Relative number	15	78	0.50	0.63 ± 0.12
Noise	12	75	0.50	0.50 ± 0.11
Social				
Social cue	12	77	0.50	0.56 ± 0.13
Gaze perception	8	89	0.50	0.56 ± 0.18
Inhibition				
A-not-B	5	86	0.33	0.37 ± 0.33
Cylinder	10	119	NA	0.54 ± 0.30

## Discussion

Here, we tested the SIH at the individual level within a group of free-ranging Japanese macaques, examining the relationship between each individual’s social centrality and their performance in cognitive tasks spanning various domains. We developed two main predictions based on the SIH, and one of them was supported but the other was not; thus, our results partially support the SIH.

Our first prediction about the link between the monkeys’ social centrality and their social cognitive ability was not supported. We predicted that the macaques with higher social centralities, especially regarding the size of their grooming networks, would show better performances in social domain tasks, as several neuroanatomical studies suggested a positive correlation between the size of individuals’ social networks and brain volumes associated with social cognition^17,26–28,32^. However, we found no relationship between measures of sociality and performance in physical or social cognition tasks. This remained true for separate analyses of tasks simulating cooperative and competitive social situations. These results indicate that occupying socially central positions may not enhance socio-cognitive abilities, such as interpreting social cues or perceiving gaze directions.

Our second prediction about the positive link between the monkeys’ social centrality and their inhibitory control was supported, although it was only eigenvector centrality that was associated with the inhibitory task performance. Interestingly, when analyzing each task separately, we found that it was only the performance of the cylinder task that showed an association with eigenvector centrality. The cylinder task and A-not-B task assess different components of inhibitory control, with the former requiring behavioral inhibition and the latter requiring cognitive inhibition^40^. This suggests that behavioral inhibition plays a crucial role in enhancing social integration and status among individual Japanese macaques. It is noteworthy that various previous studies have pointed out the link between inhibition of impulsive behavior and individual sociality. In humans, those who showed better inhibition of impulsiveness during childhood were more likely to achieve social success in the future^44,45^. Conversely, social isolation has been suggested to impair inhibitory control^46^. In non-human animals, such as Australian magpies^15^ and spotted hyenas^16^, individuals raised in larger groups showed enhanced performance in the cylinder task. Additionally, performance of the cylinder task has been associated with larger song repertoire size in song sparrows (*Melospiza melodia*)^47^ and higher frequency of cooperation in zebra finches (*Taenyopigia guttata*)^48^. Our results provide further evidence to the growing body of literature demonstrating the link between behavioral inhibition and individual sociality.

Analyzing male and female networks separately, we found that higher eigenvector centrality in both sexes correlated with better inhibitory control, particularly on the cylinder task performance. Thus, occupying socially central positions is associated with better behavioral inhibition for both males and females, even within the same-sex network. This finding is particularly notable for males, the dispersing and typically less sociable sex, since it aligns with a recent study highlighting the importance of occupying central positions within their same-sex social network. In that study, the authors found that male-immigration success was positively associated with higher eigenvector centrality values^49^. Although the dynamics of male-male social bonds are less studied compared to those of females, our findings suggest that behavioral inhibition may relate to variations in male sociality and the potential benefits arising from them.

Among the five social centralities considered in the statistical models, only eigenvector centrality explained the performance of inhibitory control tasks, and, in particular, the cylinder task. To achieve higher eigenvector centrality, individuals must have strong bonds with numerous partners and/or maintain a strong connection with other highly central individuals. Thus, achieving higher eigenvector centrality might potentially involve a more intricate social understanding, such as tracking other individuals’ social networks. Another possibility is suggested by a simulation study which showed that higher eigenvector centrality may result from a simple behavioral strategy, like increasing the number of interaction partners^50^. However, our GLMMs showed that the number of grooming partners (degree) did not explain the task performance, while eigenvector centrality did. Additionally, degree and eigenvector centrality were not highly correlated in the mixed-sex and female-network models, indicating that each of them represented different aspects of individual sociality. These results suggest that this simple behavioral rule might not be enough to explain how macaques attain higher eigenvector centrality. Therefore, behavioral inhibition may enable individuals to navigate their social environments flexibly and thereby occupy more central network positions concerning both direct and indirect connections. Further study is needed to investigate how individuals attain socially central positions from the perspective of eigenvector centrality.

Taken together, our results provide partial additional support for the SIH by demonstrating the association between inhibitory control task performance and a type of social centrality at the individual level. This should be further investigated from several viewpoints in the future studies. Firstly, the proximate mechanisms underlying the relationship between social centralities and inhibitory control remain unclear. Individual variations in social centralities may emerge from the variations in social strategies adopted by each individual in daily social interactions^50^. Accordingly, one possibility is that higher inhibitory control enables animals to engage in social interactions more efficiently, for example, by allowing them to use appropriate social signals in different contexts^16,36,51^. Secondly, we did not examine developmental backgrounds of the observed relationship between behavioral inhibition and eigenvector centrality, since we collected data solely on adult macaques, and excluded infants, juveniles or subadults. Japanese macaques broaden their social network throughout the developmental stages, largely influenced by the social relationship of their mothers at the early stage but later forming connections with reduced maternal influence^52,53^. Furthermore, it is worth mentioning here that we observed a significant decrease of the A-not-B task performance of the oldest individuals in the mixed-sex network (see supplemental Table S3). This suggests that Japanese macaques may experience a decline in inhibitory control thorough aging, especially in cognitive inhibition. Considering that older macaques are more likely to experience social isolation within their social group^54,55^, there might be a link between decreased inhibitory control ability and social isolation, which is mediated by aging. Further investigation of the link between cognitive factors and sociality with subjects of different age stages may reveal how this relationship can emerge through development and aging. Thirdly, only a few studies have directly tested the fitness consequences of cognition^15,56^. However, since the SIH was originally developed to explain the circumstances favouring the evolution of greater cognitive capacities, more empirical research on the relationship between cognition and individual fitness is needed. Considering that some studies have reported a positive relationship between higher social centralities and fitness among primates^5,57^, it is plausible that greater cognitive abilities could indirectly lead to greater reproductive success through enhanced social centralities. Lastly, the relationship between sociality and cognition could differ depending on the nature of the group. One promising approach in this context is to compare the species or groups with varying degrees of social tolerance. Recent evidence suggests that species exhibiting higher social tolerance outperform less tolerant ones in certain cognitive domains, particularly those related to communication and cooperation^36,58,59^. Japanese macaques would be an important model species in this line of research; they exhibit significant inter-group differences in social tolerance levels^60–62^, which provides an ideal opportunity for inter-group comparisons. Therefore, inter-species and inter-group comparisons within species could provide deeper insights into the evolutionary mechanisms of cognitive abilities among primates.

To our knowledge, this is the first evidence of a link between social centrality, specifically eigenvector centrality, and inhibitory control ability in non-human animals. The combination of our field observation and experiments with free-ranging monkeys revealed that only the inhibitory control task performance was associated with one type of social centrality, with no link found between physical or social cognition and any measures of social centrality. Our study suggests that inhibitory control, one of the executive functions related to behavioral flexibility, may predominantly underly individual variations in sociality among Japanese macaques. Testing the SIH at the individual level is an important contribution to our understanding of the origin of superior cognitive capacities. Comparative studies across various species or groups would offer valuable insights for advancing evolutionary perspectives. The combination of natural observation of animal societies and cognitive experiments conducted in their natural environment, as utilized in the current study, constitutes a powerful tool for future research on comparative cognition.

## Methods

### Study group and subjects

We conducted the present study in a free-ranging group of Japanese macaques inhabiting the southern part of Awajishima Island, Japan. This group has been artificially provisioned since 1967 by the Awajishima Monkey Center (AMC). The individuals are partially identified and observational study has been done since 1978 ^63^. The information on kin relationships among individuals of the group was only partially available at the time of the study. The group visits the feeding site almost every day, except from June to the beginning of July and September to November, when the group mainly ranges in the forest for foraging. AMC staff feed the group twice or three times a day with wheat and soybeans. The group size and composition during study periods (2017–2020 for behavioral observation and 2021 for cognitive tests: see below) were estimated to be 399 (adult males: 47; adult females: 181) in 2017, 381 (adult males: 35; adult females: 186) in 2018, 471 (adult males: 55; adult females: 223) in 2019, 454 in 2020 (adult males: 45; adult females: 227), and 474 in 2021 (adult males: 53; adult females: 241). We counted the number of individuals in the group in winter because the group size (the number of macaques that usually visit the feeding site) differs according to the season in this group and the largest size is observed in winter due to the temporal migration of peripheral males into the group for mating.

### Data collection on individual sociality

#### Observational methods

We recorded grooming as a measure of sociality. We used scan sampling method for behavioral observation, in which we walked through the feeding site on a designated route and recorded all grooming dyads of adult macaques. Since our subject group was provisioned, most of the macaques usually stayed in the vicinity of the feeding site and could easily be observed thorough out the day. In addition, we selected the route of scan sampling so that we could walk the feeding site evenly, which was the same in every scans. These enabled us to record almost all of the grooming interactions between the adult macaques. We recorded the names of the participants for each dyad. There was an interval of at least 30 min between each scan session. The observation period was 288 days, from June 2017 to March 2020, with a total of 871 sessions (average 3.1 sessions/day). We focused on the 218 adult macaques present in the group during the observational period (23 males; 195 females). Additionally, we calculated the hierarchal rank of each individual based on the results of food-dominance tests and dyadic agonistic interactions. The rank was calculated for males and females separately, and each individual was assigned one of three rank categories (i.e., low, middle, or high) according to their rank position. We used this categorized rank as an explanatory variable in the statistical models (see below). For the detailed methods of rank calculation, see the Supplementary Material S1.

#### Social network analyses

From the grooming interactions recorded, we built a symmetric matrix with rows columns representing each individual, and the number of times grooming was observed in each dyad as components, which was subsequently analyzed as a weighted grooming network. Note that we constructed one network for the analysis from approximately three years of observational data because (1) our subject group was extremely large, therefore, relatively large amount of social data was required to measure the individual social centralities, and (2) the topological structures of the network seemed stable over the years (we used quadratic assignment procedure to compare the network structures: year 1 (June 2017 – March 2018) and year 2 (April 2018- March 2019): *r* = 0.67, *p* < 0.0001; year 2 and year 3 (April 2019 – March 2020): *r* = 0.69, *p* < 0.0001; year 1 and year 3: *r* = 0.61, *p* < 0.0001). For this network, we calculated five social centrality measures that could represent the sociality of each individual in the group: i.e., degree, strength, eigenvector, betweenness and closeness centrality. We chose these centrality measures following the neuroanatomical study conducted on a free-ranging rhesus macaque group^32^. Degree indicates the number of grooming partners a macaque has had, while strength counts the total grooming interactions. Eigenvector centrality considers the strength of connections of an individual has, considering its partners’ connections to others as well. Betweenness centrality measures a node’s role in bridging network connections, and closeness centrality assesses proximity to other nodes. We calculated weighted versions of these centralities.

### Field experiments using the cognition test battery

#### Subjects

We conducted the cognitive experiments from December 2021 to September 2022. We tested 119 adult individuals whose social network data had been collected during the observation period stated above, although the number of participants varied across tasks (see Supplementary Material S1).

#### Cognitive test battery

We developed a modified version of Primate Cognition Test Battery (PCTB) consisting of eight tasks examining physical cognition (four tasks: spatial memory, transposition memory, relative number, and noise), social cognition (two tasks: social cue and gaze perception), and inhibitory control (two tasks: A-not-B and cylinder). We chose tasks used in previous studies^20,36,37,64^ that were applicable to free-ranging monkeys in an outdoor environment (see Table 1 and Supplementary Material S1 for details of each task).

**Fig. 3:**
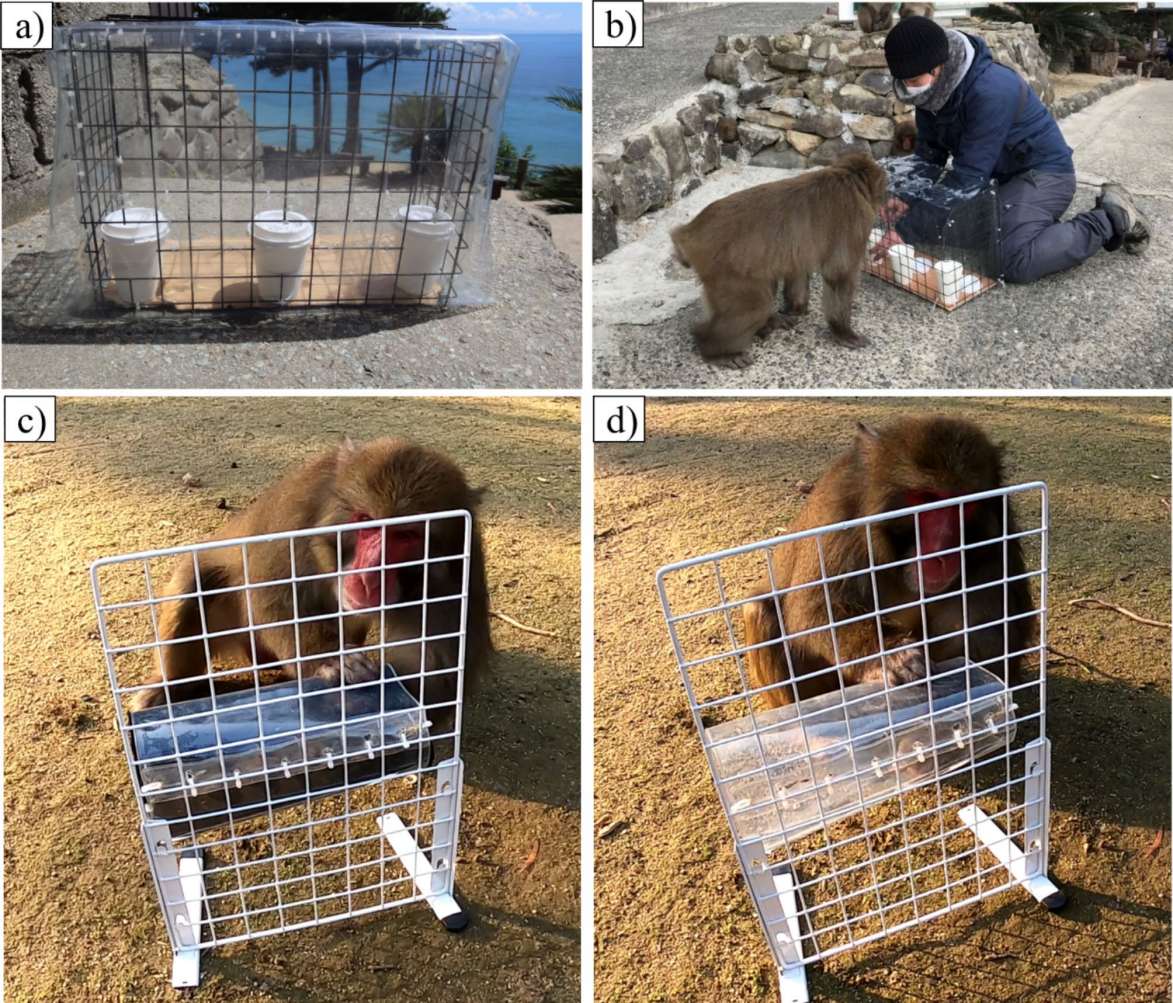
The appratuses used in the filed experiment. (**a**): The apparatus used for the tasks involving an object-choice paradigm (spatial memory, transposition, noise, relative number, pointing, A-not-B). The front side of the apparatus was covered by a transparent sheet so that subjects could not touch the objects until the experimenter opened it; (**b**): Field experiment using the apparatus. The subject sat in front of the appratus; (**c**): The apparatus used for the cylinder task with the opaque cylinder (familiarization trial); (**d**): the cylinder task apparatus with the transparent cylinder (test trials).

We tested each subject in the following order: cylinder, A-not-B, spatial memory, transposition, relative number, social cue, and noise. Regarding the gaze perception task, we completed it flexibly when possible, without adhering to the predetermined task order, as it required some assistants in the experiment. Since our subjects lived in a free-ranging group, we created portable experimental apparatuses so that we could conduct the whole experiment in the outdoor environment (Fig. 3). YK conducted all the experimental trials, which were recorded by a video camera (GoPro HERO 10 Black) attached on his chest. YK later coded the subjects’ responses from the videos. As a food reward, we used pieces of sweet potato except for the relative number task, in which we used soybeans instead (see Supplementary Material S1 for details of the experimental procedure).

### Statistical analysis

We used generalized linear mixed models (GLMMs) with binomial error structure and logit link function to analyze what factors contributed to the success of the cognitive tasks. We fitted the models first at the domain levels (i.e., physical, social and inhibitory), and then fitted at the task levels. We conducted the task-level analyzes because each task was considered to represent different aspects of cognition even within the same domain. The response variables were trial response (success/failure) in all the models. The explanatory variables included ‘sex’, ‘dominance rank category’, ‘age’ (three categories: adult, 6–19 years old; old, 20–24 years old; and very old: 25 or older), and five types of social centralities (‘degree’, ‘strength’, ‘eigenvector centrality’, ‘betweenness centrality’, ‘closeness centrality’). At the domain-level analyses, we also included ‘task’ as an explanatory variable, to see whether the performance of the macaques differed between tasks within the same domain. Task-level models included the same explanatory variables as the domain-level models, except that ‘trial number’ instead of ‘task’ was added as an explanatory variable. All the social centrality measures were rescaled to range from a minimum value of 0 to a maximum value of 1.

We analyzed social centrality for mixed, male-only, and female-only networks of Japanese macaques and fitted GLMMs separately (defined as *mixed-network*, *male-network* and *female-network model*). Thus, we built 33 models in total. We included the individual ID as a random variable in all the models to control for pseudo replication. This approach considers both within and between different sex social bonds, providing a comprehensive understanding of their association with cognitive abilities.

We assessed the variance inflation factors (VIFs) for each model to deal with multicollinearity problems. The variables with the highest values of VIFs were systematically removed from the model until all the variables included had VIFs lower than five. Following this procedure, ‘strength’ was removed from all domain and task level models for mixed- and female-network models. For male-network models, ‘degree’, ‘strength’, ‘betweenness’ were removed in all models, and ‘closeness’ and ‘age category’ were removed specifically in the two inhibitory control tasks.

All analyses were conducted using R ver. 4.0.3. We fitted GLMMs using the package lme4 ^65^. We estimated the parameters by maximum likelihood and using a Laplace approximation. We constructed null models that contained only the intercept and the random factor as explanatory variables and conducted likelihood ratio tests (LRT) to test the significance of the full models. We showed the results of GLMMs only when significant differences between full and null models were found. We used the R package igraph^66^ to compute the social centralities and used the lrtest function in the lmtest package^67^ to conduct LRT.

### Ethics statements

This study received approval from the Animal Experimentation Committee of the Wildlife Research Center of Kyoto University (ethical approval number: WRC-2021–019 A). We declare that all experiments and behavioral observations were conducted following the regulation on Animal Experimentation at Kyoto University. No subjects were physically constrained or separated from the group during cognitive experiments, and they were completely free to participate in and leave the experiments. Normal daily provisioning was conducted during the whole study period.

## Electronic Supplementary Material

Below is the link to the electronic supplementary material.

Supplementary Material 1

Supplementary Material 2

## Data Availability

Data supporting the current results is provided within the supplementary information files.
