# Supplementary material for: Higher eigenvector centrality in grooming network is linked to better inhibitory control task performance but not other cognitive tasks in free-ranging Japanese macaques
Source: Sci Rep. 2024 Nov 19;14:26804. doi: 10.1038/s41598-024-77912-7 (PMC11577106; doi:10.1038/s41598-024-77912-7)
Supplement: Supplementary file 1 — Supplementary Material 1 [file 41598_2024_77912_MOESM1_ESM.docx]

**Supplementary material S1**

Article: Higher eigenvector centrality in grooming network is linked to better inhibitory control task performance but not other cognitive tasks in free-ranging Japanese macaques

Yu Kaigaishi*^1^, Shinya Yamamoto^1,2^

1. Kyoto University Institute for Advanced Study

2. Wildlife Research Center, Kyoto University

**Methods**

Hierarchical rank calculation

To calculate the dominance rank of each adult macaque, we recorded the results of food-dominance tests and dyadic agonistic interactions (aggression and supplant) for the 23 males and 196 adult females. In food-dominance tests, we dropped a piece of food (soybean) between two individuals sitting 1-2 m apart and recorded who took it. When one individual took the food and the other did not show any attempts to take it, we recorded the former as the dominant and the latter as the subordinate. If both individuals tried to take the food, or one took the food and soon ran away from there, we did not record the results of the test because the determination of dominance relationships could be less straightforward in such cases. We used ad lib sampling method to record the dyadic agonistic interactions between two individuals. We also included those interactions that were recorded during focal observations in the analysis of dominance hierarchy. We defined the aggressor as the dominant, and the victim as the subordinate. We did not include those interactions in the analysis in which the victim showed counter-aggression toward the aggressor. Supplant was defined as when one individual approached another within 2 m, the approached individual moved away from the place without receiving any aggression. We recorded the individual that approached the other as the dominant, and that left the place as the subordinate. In the present study, we recorded 590 interactions for males (agonistic interaction: 26; food-dominance test: 564) and 14270 interactions for females (agonistic interaction: 657; food-dominance test: 13613).

Of all the possible combinations, 98 pairs (38.7%) for males and 7942 pairs (41.5%) for females were recorded at least once. Due to the large proportion of unknown relationships in our dataset, we used the percolation conductance method to explore the hierarchical rank structure^1^. This method uses the results of direct dominance interactions (here, agonistic interactions and food-dominance tests) to generate the indirect pathways of the win/loss network among individuals. For example, if we observed that individual A dominated individual B and individual B dominated individual C, then we could gain the indirect pathway between A and C as A > B > C. In this method, dominance relationships are estimated using combinations of direct and multiple indirect pathways between the two individuals, by calculating dominance probabilities between them. Thus dominance probabilities could be calculated for those individuals in which direct dominance interactions were not recorded. Therefore, the use of this method allowed us to increase the sample size and improve the estimation accuracy, owing to the inclusion of indirect pathways^2^. We used the R package Perc^3^ to conduct the percolation conductance method. We set the number of repeated random simulations as 10000, and the maximum distance of indirect pathways as 4 due to the limitation of the machine power.

Experimental procedures

At the beginning of testing period (December 2021), there were 194 adult individuals present in the group (17 males; 177 females). These individuals were considered as *potential test subjects*, and, instead of choosing which to test with a priori, we conducted the experiments with any of them depending on the situation (see below). Since our subject was a free-ranging group and we had to conduct the experiments in the outdoor field, there were always possibilities of interference from the individuals other than the one we wanted to test with. Thus, we carried out each trial when we found one of the potential test subjects being in isolation from other individuals or when the subject was the most dominant one around there. When interfered by other individuals during testing, we suspended the task at that point. The tests were resumed from that point when we found the appropriate situation again. Due to this experimental design, the number of subjects differed across tasks to some extent (Table 1).

Since our subjects lived in a free-ranging group, we created portable experimental apparatuses so that we could conduct the whole experiment in the outdoor environment. For the tasks except for the cylinder and the gaze perception, we used object-choice paradigm. We used a wire mesh basket as an experimental apparatus (Fig. S1). The side facing the experimenter was fully open so that he could have manipulated the objects during the tests. The side facing the macaques was cover by wire mesh but had three holes so that the macaques could have touched the objects to make choice. Since the sides of the basket was covered with transparent plastic sheet, the macaques could watch the whole manipulation procedure but could not touch the objects until the experimenter opened the sheet and allowed them to choose. We used paper cups with lids to hide rewards inside. For the cylinder task, we used a plastic cylinder attached to a portable wire net (Fig. 1). There were two types of cylinders, opaque or transparent, which were used according to the testing condition (see below for the testing procedure). The two cylinders were identical except that the opaque one was covered by black cloth. The opaque cylinder was not attached to the wire net but could be inserted into the transparent one, so that we could smoothly change the testing condition in the outdoor experimental environment.

Before first participating in the task using this apparatus (i.e., A-not-B task), each subject experienced a familiarization process. First, the experimenter placed the apparatus in front of the subject. Then, he put a single open cup in front of one of the three holes of the apparatus and baited it, so that the subjects could take the reward directly from the cup thorough the hole. This procedure was repeated three times, the cup being put in front of the different positions of holes (middle, left, or right) each time. In the next step, the experimenter closed the cup with a lid after baiting, while the front side of the apparatus was closed with a plastic sheet so that the macaques could not touch the cup. Then, he opened the sheet and allowed them to touch the closed cup. Once they touched it, he opened the cup and let them take the reward from the inside. This procedure was repeated six times, and the position of the cup was changed each time.

Details of the tasks composing our Primate Cognitive Test Battery (PCTB)

Our PCTB consisted of a physical domain (four tasks), a social domain (two tasks), and an inhibitory domain (two tasks) based on several previous studies^4, 5, 6^ with some modification to fit for the field environment and target species. We selected the four tasks of the physical domain to cover all the three scales that was proposed in previous studies ^4, 5, 6, 7^: space, quantities, causality scales. The space scale involved spatial memory and transposition task, which respectively tested monkey’s ability to remember the position of cups containing the rewards or keep track of the trajectory of the baited cups. The relative number task belonged to the quantities scale, which tested whether monkeys distinguished different numbers of food rewards. Lastly, in the causality scale, we adopted the noise task to test the macaque’s understanding of the causal relationships between the presence of food reward and the sound produced from the shaken cups.

We selected two tasks of the social domain to cover the communication and theory of mind scales (we do not believe the term “theory of mind” is appropriate in our study, but still use this term to accord with the previous studies), two out of the three scales proposed in the previous study^7^, although we have eliminated the social learning scale since it is considered to be too difficult for this species^4^. The communication scale was measured in a social cue task, where the macaques were tested whether they could choose the correct cups following the human experimenter’s pointing gestures. The theory of mind scale was measured by gaze perception task which tested the macaques’ understanding of human gaze directions, by presenting them with a situation in which they had to take food from the human facing backward. We adopted these two tasks since the former represented socially cooperative situations while the latter represented socially competitive ones.

We selected two tasks, A-not-B and cylinder tasks, of the inhibitory domain to cover the two types of inhibition (i.e., cognitive inhibition and behavioral inhibition) respectively^8^. In the A not B task, the macaques were first trained to choose one of three cups which was placed always the same side (left or right). Then, in the test condition, food was hidden in the cup placed in the opposite side, and hence they had to choose the different cup while inhibiting their propensity to take the incorrect cup they had learned to. In the cylinder task, first the macaques learned to take the reward from an opaque cylinder by inserting their hands from the side. Then, in the test condition, the cylinder became transparent and the food inside was visible to them. Therefore, they had to inhibit their impulsiveness to directly taking the food by directly reaching the cylinder but to insert their hands as they had learned.

*Physical domain*

**Spatial memory.** The experimenter hid food rewards in two of the three cups in full view of the subjects. Then, he opened the plastic sheet so that the subjects could choose one cup by touching it though the hole of the basket. Once they made choice, he opened the cup and allowed them to see the inside. If they made a correct choice, they could take the reward from the cup and were allowed to make another choice. If incorrect, the experimenter closed the sheet so that they could not make any other choice. The trial was considered as successful when the subjects chose both of the two correct cups. This task consisted of six trials, and which cups were baited was randomized between trials.

**Transposition.** In the transposition task, the experimenter first hid a food in one of the three cups. Then, he changed the position of the baited cup with the other cups in three different ways: (1) the baited cup was switched with one of the two empty cups; (2) the baited cup was switched with the empty cups twice (3) the baited cup was switched with one of the empty cups, and then the positions of the two empty cups were switched. The subjects watched all the manipulations, and were allowed to make a choice once the manipulation procedure was completed and the plastic sheet was opened. They gained the reward only when they could choose the correct cup, and the trial was recorded as successful. This task consisted of six trials, and which cup was baited and switched was randomized. They experienced each condition two times, in the order of condition1, 2, and 3.

**Relative number.** The experimenter placed two plates in the middle of the apparatus and covered it with a black cloth to prevent the subjects from watching the baiting procedure. He put different amounts of food rewards (we used soybeans here, so that the sizes can be easily controlled) on each plate, and then removed the cloth and showed the plates to the macaques. He then moved the places to the right and left side of the apparatus, and opened the sheet to allow the macaques to choose one plate. Once they made a choice, the experimenter immediately retrieved the other plate so that they could not take it. Each subject experienced one trial for each of the following pairs of numbers: *1:2, 1:3, 1:4, 1:5, 2:3, 2:4, 2:5, 2:6, 3:4, 3:5, 3:6, 3:7, 4:6, 4:7, 4:8* (note that the order and which side was more rewarded were randomized between and within subjects). This task consisted of 15 trials and success was recorded when subjects chose the larger reward. Which side of the plated was baited more was randomized and counter-balanced between trials.

**Noise.** The experimenter placed two cups in the apparatus, took both and put them behind his back to bait one of the cups, so that the macaques could not see the baiting procedure. Then he again put them in the apparatus and performed the experimental manipulation following one of the two procedures below. In the *Noise full* condition, the experimenter shook the cup containing food three times while only lifted the empty one without shaking it. In the *Noise empty* condition, by contrast, he shook the empty cup three times while only lifted the baited one. Whether he started with the baited or empty cup was randomized in both conditions. After this manipulation, he opened the plastic sheet on the apparatus and allowed the subjects to choose one of the cups. They gained the reward when they choice the baited cup, the trial which was considered as successful. They could make only one choice in each trial. This task consisted of 12 trials, and which side of the cup was baited and which conditions they experienced was randomized and counter-balanced between trials.

*Social domain*

**Social cue.** As in the noise task, the experimenter first baited one of the cups behind his back to prevent the subjects from seeing the baiting procedure. Then, he placed the cups on the sides of the apparatus, and touched the baited one with the extended index finger. He then opened the sheet and allowed them to choose one of them. They gained the reward when they chose the baited cup, and could make only one choice in each trial. The trial was successful when they chose the correct one. This task consisted of 12 trials, and which cup was baited was randomized and counter-balanced between trials.

**Gaze perception.** We followed the procedure of Flombaum and Santos ^9^’s experiment 1, but slightly modified it by adding a familiarization phase. In this task, we needed two more experimenters in addition to the main experimenter (YK). Before starting the test phase, we introduced a familiarization phase to allow the monkeys to learn that they could not take a reward when an experimenter was facing toward them. In this phase, one of the experimenters other than YK sat in front of the subjects 3-5 m apart facing toward them, with a plate placed in front. YK then put a food reward on the plate. When the subjects approached and tried to take the reward, the experimenter took the plate and stood up so that they did not allow them to take it. Before the test phase, the subjects experienced this procedure 2 times, and faced with different experimenters each time. In the test phase the two experimenters other than YK sat apart from the subjects 3-5 m apart. One of them was facing forward while the other backward, and plates were placed on the side facing the subjects (in front of the one facing forward and behind the one facing backward). The distance between the experimenters was approximately 180 cm. YK placed the rewards simultaneously on each plate and immediately moved straight away from the subjects, so that his movements did not affect the subjects’ route choices. When the subjects approached the experimenter facing forward, he/she took the plate and stood up so that they did not allow them to take it. When they approached the one facing backward, both experimenters did not move and allowed them to take the reward. The trial was considered as successful when they choice the experimenter facing backward. This task consisted of 8 trials, and the side of the experimenters and which faced forward/backward was randomized between trials. Note that the experimenters other than YK was naïve to the macaques, i.e., they had never visited AMC until this experiment was conducted.

*Inhibitory domain*

**A-not-B.** This task consisted of pretest and test phases, following MacLean et al.^10^. In the pretest, the experimenter put a reward in the cup placed on either the right or left side in full view of the subjects. He then opened the sheet and allowed the macaques to choose one cup. When they made a choice, the experimenter opened the cup so that they could see the inside. If they choice the correct one, they could take the reward. Then, the sheet was closed and the same procedure was repeated. Note that the same side always baited in the pretest condition. Subjects had to succeed in five consecutive trials to proceed to the test condition. In the test condition, he first put the reward in the same side as they had experienced in the pretest, but then he opened the baited cup and moved the reward to the cup on the opposite side. Then, he opened the sheet and allowed the subjects to make a choice. The trial was considered as successful when the subject choice the correct cup. This task consisted of five trials, and the side baited in the pretest was randomized between the subjects.

**Cylinder.** This task consisted of the pretest and the test phases. In the pretest condition, the experimenter put a reward in a cylinder covered with black cloth (i.e., the cylinder was opaque and the macaques could not see the inside) in full view of the subjects. They had to put their hands into the cylinder through the sides to take the reward, which was consecutively repeated five times. Then, in the test phase, the procedure and the apparatus were the same except that the cylinder was transparent and they could see the inside. The trial was considered as successful when the subjects took the reward without touching the cylinder, and unsuccessful when they touched the cylinder before taking out the reward (regardless of whether they finally gained the reward). This task consisted of 10 trials.

Results

Physical domain

In the mixed-sex model, only the type of the tasks explained the probability of success in a trial, suggesting that they performed better on the relative number (*z* = 0.49, *p* < 0.01) but worse on the spatial memory (*z* = -2.49, *p* = 0.01) and transposition (*z* = -4.85, *p* < 0.01) compared to the noise task (Table S1). There were no effects of social centralities on the task performances. Similarly, only the task type explained the trial success in both male- and female-network models. The males performed better on the relative number (*z* = 2.75, *p* = 0.01) but worse on the transposition task (*z* = -2.39, *p* = 0.02), the females performed better on the relative number (*z* = 4.82, *p* < 0.01) but worse on the spatial memory (*z* = -2.45, *p* = 0.01) and transposition (*z* = -4.33, *p* < 0.01) compared to the noise task (Table S1).

At the task levels, any of the models did not explain the task performances better than the null models (spatial memory: *χ*^2^ = 7.65, df = 10, *p* = 0.66; transposition: *χ*^2^ = 6.77, df = 10, *p* = 0.75; noise: *χ*^2^ = 12.70, df = 10, *p* = 0.24; *χ*^2^ = 4.34, df = 10, *p* = 0.93). This was also the case for the male-network (spatial memory: *χ*^2^ = 2.19, df = 6, *p* = 0.90; transposition: *χ*^2^ = 3.98, df = 6, *p* = 0.68; relative number: *χ*^2^ = 8.02, df = 6, *p* = 0.24; noise: *χ*^2^ = 4.54, df = 6, *p* = 0.60) and the female-network models (spatial memory: *χ*^2^ = 7.63, df = 9, *p* = 0.57; transposition: *χ*^2^ = 5.68, df = 9, *p* = 0.77; relative number: *χ*^2^ = 2.98, df = 9, *p* = 0.97; noise: *χ*^2^ = 8.73, df = 9, *p* = 0.46). See Table S2 for the detailed GLMM results.

Inhibitory domain

In this domain, the effects of the task and eigenvector centrality were significant in the mixed-sex (task: *z* = 7.45, *p* < 0.01; eigenvector centrality: *z* = 3.16, *p* < 0.01) and female-network model (task; *z* = 7.37, *p* < 0.01; eigenvector centrality: *z* = 2.79, *p* < 0.01). In the male-network model, only the effect of eigenvector centrality was significant (*z* = 3.27; *p* < 0.01; Table S5). When we analyzed each task separately, we found that the full models were a better fit than the null models for both tasks, in the mixed-sex (A-not-B: *χ*^2^ = 38.13, df = 10, *p* < 0.01; cylinder: *χ*^2^ =87.71, df = 10, *p* < 0.01), male-network (A-not-B: *χ*^2^ = 22.55, df = 7, *p* < 0.01; cylinder: *χ*^2^ = 15.13, df = 4, *p* < 0.01) and female-network model (A-not-B: *χ*^2^ = 29.08, df = 9, *p* < 0.01; cylinder: *χ*^2^ = 80.31, df = 9, *p* < 0.01). At the task level, only trial number was significantly associated with the task success (mixed-sex: *z* = 4.46, *p* < 0.01; male: *z* = 2.24, *p* = 0.04; female: trial: *z* = 3.88, *p* < 0.01; table S6). On the other hand, both trial and eigenvector centrality had significant effects in the cylinder task (mixed-sex: trial: *z* = 7.34, *p* < 0.01; eigenvector centrality *z* = 3.27, *p* < 0.01; male: trial: *z* = 2.00, *p* = 0.045; eigenvector centrality: *z* = 3.00, *p* < 0.01; female: trial; *z* =7.01, *p* < 0.01; eigenvector centrality: *z* = 2.84, *p* < 0.01; Table S6). These results indicate that the effect of eigenvector centrality value was associated particularly with the cylinder task performance.


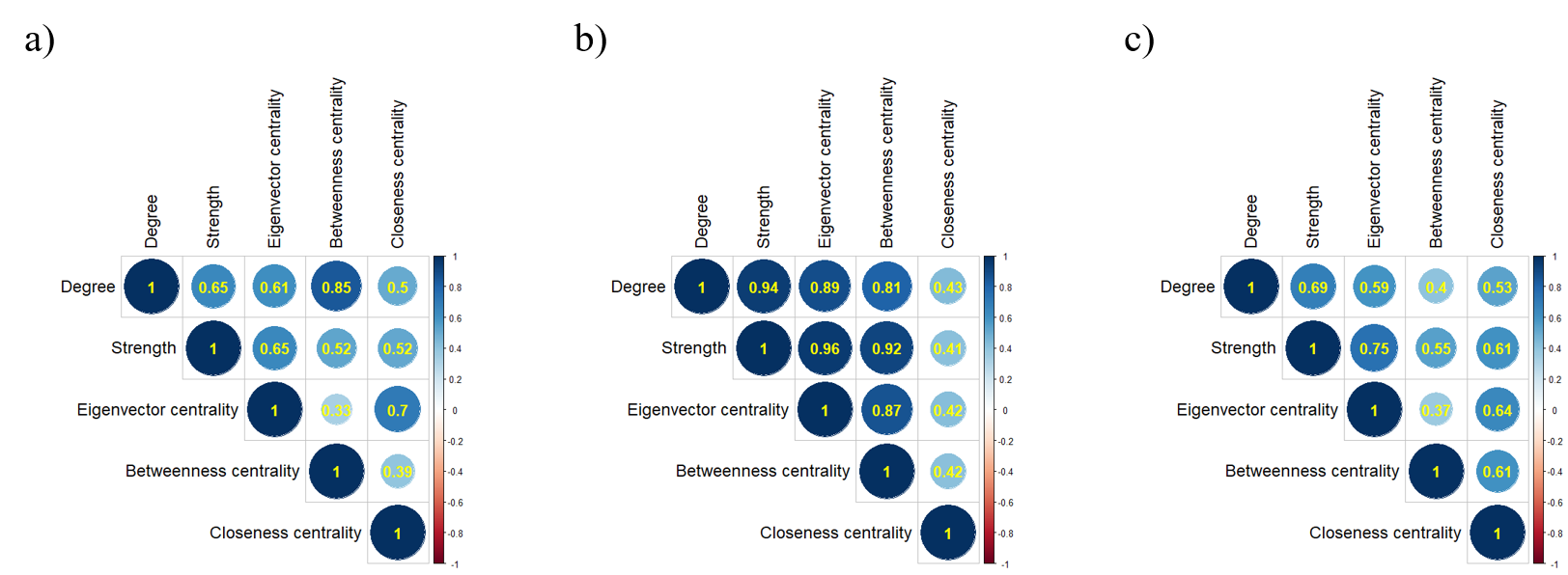


Fig. S1. The correlation matrices between the 5 social centralities. (a): mixed-sex network; (b): male-network; (c): female-network.

Table S1. The results of GLMMs in the physical domain.

| *Physical domain* | **Mixed-network model** | | | | | | | | | | | | | | | | | | | |  | | |  | **Male-network model** | | | | | | | | | | | | | | | | | | | |  | | **Female-network model** | | | | | | | | | | | | | | | | | |
| --- | --- | --- | --- | --- | --- | --- | --- | --- | --- | --- | --- | --- | --- | --- | --- | --- | --- | --- | --- | --- | --- | --- | --- | --- | --- | --- | --- | --- | --- | --- | --- | --- | --- | --- | --- | --- | --- | --- | --- | --- | --- | --- | --- | --- | --- | --- | --- | --- | --- | --- | --- | --- | --- | --- | --- | --- | --- | --- | --- | --- | --- | --- | --- | --- |
| Parameter | Coefficient | | | CI_low | | | CI_high | | | | z value | | | | Pr (>\|z\|) | | | | |  | | |  | | Coefficient | | | | CI_low | | | | CI_high | | | | z value | | | | Pr (>\|z\|) | | | |  | | Coefficient | | | | CI_low | | | | CI_high | | | | z value | | | Pr (>\|z\|) | | |
| (Intercept) |  |  | 0.39 |  | - | 0.08 | |  |  | 0.86 | |  |  | 1.61 | |  |  | 0.11 |  | | |  | | | |  |  | 0.47 | |  | - | 0.76 | |  |  | 1.70 | |  |  | 0.74 | |  |  | 0.46 | |  | |  |  | 0.43 | |  | - | 0.02 | |  |  | 0.88 |  |  | 1.87 |  |  | 0.06 |
| sex [male] |  | - | 0.18 |  | - | 0.45 | |  |  | 0.10 | |  | - | 1.27 | |  |  | 0.21 |  | | |  | | | |  |  | ‒ | |  |  | ‒ | |  |  | ‒ | |  |  | ‒ | |  |  | ‒ | |  | |  |  | ‒ | |  |  | ‒ | |  |  | ‒ |  |  | ‒ |  |  | ‒ |
| rank [low] |  | - | 0.11 |  | - | 0.42 | |  |  | 0.20 | |  | - | 0.67 | |  |  | 0.50 |  | | |  | | | |  |  | 0.02 | |  | - | 0.97 | |  |  | 1.01 | |  |  | 0.03 | |  |  | 0.97 | |  | |  | - | 0.13 | |  | - | 0.47 | |  |  | 0.21 |  | - | 0.74 |  |  | 0.46 |
| rank [mid] |  | - | 0.12 |  | - | 0.35 | |  |  | 0.12 | |  | - | 0.98 | |  |  | 0.33 |  | | |  | | | |  |  | 0.39 | |  | - | 0.30 | |  |  | 1.08 | |  |  | 1.11 | |  |  | 0.27 | |  | |  | - | 0.16 | |  | - | 0.41 | |  |  | 0.08 |  | - | 1.29 |  |  | 0.20 |
| age category [old] |  | - | 0.11 |  | - | 0.33 | |  |  | 0.11 | |  | - | 0.98 | |  |  | 0.32 |  | | |  | | | |  |  | 0.09 | |  | - | 0.78 | |  |  | 0.96 | |  |  | 0.20 | |  |  | 0.84 | |  | |  | - | 0.11 | |  | - | 0.36 | |  |  | 0.14 |  | - | 0.88 |  |  | 0.38 |
| age category [very old] |  | - | 0.09 |  | - | 0.22 | |  |  | 0.40 | |  | - | 0.56 | |  |  | 0.57 |  | | |  | | | |  |  | ‒ | |  |  | ‒ | |  |  | ‒ | |  |  | ‒ | |  |  | ‒ | |  | |  |  | 0.11 | |  | - | 0.21 | |  |  | 0.43 |  |  | 0.68 |  |  | 0.50 |
| task [relative number] |  |  | **0.49** |  | **-** | **0.32** | |  |  | **0.67** | |  |  | **5.47** | |  |  | **0.00** |  | | |  | | | |  |  | **0.73** | |  |  | **0.21** | |  |  | **1.25** | |  |  | **2.75** | |  |  | **0.01** | |  | |  |  | **0.46** | |  | - | **0.28** | |  |  | **0.65** |  |  | **4.82** |  |  | **0.00** |
| task [spatial memory] |  | - | **0.28** |  | **-** | **0.51** | |  | **-** | **0.06** | |  | **-** | **2.49** | |  |  | **0.01** |  | | |  | | | |  | - | 0.19 | |  | - | 0.86 | |  |  | 0.48 | |  | - | 0.57 | |  |  | 057 | |  | |  | - | **0.30** | |  | - | **0.53** | |  | - | **0.06** |  | **-** | **2.45** |  |  | **0.01** |
| task [transposition] |  | - | **0.56** |  | **-** | **0.79** | |  | **-** | **0.33** | |  | **-** | **4.85** | |  |  | **0.00** |  | | |  | | | |  | **-** | **0.88** | |  | **-** | **1.60** | |  | **-** | **0.16** | |  | **-** | **2.39** | |  |  | **0.02** | |  | |  | - | **0.53** | |  | - | **0.77** | |  | - | **0.29** |  | **-** | **4.33** |  |  | **0.00** |
| degree |  | - | 0.25 |  | - | 0.73 | |  |  | 0.24 | |  | - | 1.00 | |  |  | 0.32 |  | | |  | | | |  |  | ‒ | |  |  | ‒ | |  |  | ‒ | |  |  | ‒ | |  |  | ‒ | |  | |  | - | 0.31 | |  | - | 0.85 | |  |  | 0.233 |  | - | 1.13 |  |  | 0.26 |
| eigenvector |  | - | 0.09 |  | - | 0.63 | |  |  | 0.45 | |  | - | 0.31 | |  |  | 0.75 |  | | |  | | | |  | - | 0.17 | |  | - | 1.36 | |  |  | 1.02 | |  | - | 0.27 | |  |  | 0.78 | |  | |  | - | 0.12 | |  | - | 0.70 | |  |  | 0.45 |  | - | 0.42 |  |  | 0.67 |
| betweenness |  | - | 0.16 |  | - | 0.42 | |  |  | 0.74 | |  | - | 0.54 | |  |  | 0.59 |  | | |  | | | |  |  | ‒ | |  |  | ‒ | |  |  | ‒ | |  |  | ‒ | |  |  | ‒ | |  | |  |  | 0.17 | |  | - | 0.55 | |  |  | 0.89 |  |  | 0.46 |  |  | 0.64 |
| closeness |  |  | 0.26 |  | - | 0.97 | |  |  | 0.44 | |  | - | 0.73 | |  |  | 0.46 |  | | |  | | | |  | - | 1.70 | |  | - | 3.73 | |  |  | 0.33 | |  | - | 1.64 | |  |  | 0.10 | |  | |  | **-** | 0.27 | |  | - | 1.00 | |  |  | 0.46 |  | - | 0.73 |  |  | 0.46 |

All models were built using binomial generalized linear mixed models with a log link function and included random effect of Japanese macaque identity (ID). The mixed-network model included social centrality variables calculated within a network that included both males and females. The male-network model derived centrality variables exclusively from male-male social network, and the female-network model did so from female-female social network. Bold values indicate significant effects. For sex, rank, and age category, the reference categories were “female”, “high”, and “adult”, respectively.Table S2. The results of GLMMs in the spatial memory, transposition, relative number task, and noise task.

| *Spatial memory* | **Mixed-network model** | | | | | | | | | | | | | | | | | | | |  | **Male-network model** | | | | | | | | | | | | | | | | | | | |  | | **Female-network model** | | | | | | | | | | | | | | | | | | |  |
| --- | --- | --- | --- | --- | --- | --- | --- | --- | --- | --- | --- | --- | --- | --- | --- | --- | --- | --- | --- | --- | --- | --- | --- | --- | --- | --- | --- | --- | --- | --- | --- | --- | --- | --- | --- | --- | --- | --- | --- | --- | --- | --- | --- | --- | --- | --- | --- | --- | --- | --- | --- | --- | --- | --- | --- | --- | --- | --- | --- | --- | --- | --- | --- |
| Parameter | Coefficient | | | CI_low | | | CI_high | | | | z value | | | | Pr (>\|z\|) | | | | |  | | Coefficient | | | | CI_low | | | | CI_high | | | | z value | | | | Pr (>\|z\|) | | | |  | | Coefficient | | | | | CI_low | | | | CI_high | | | | z value | | | Pr (>\|z\|) | | |  |
| (Intercept) |  |  | 0.50 |  | - | 0.70 | |  |  | 1.70 | |  |  | 0.82 | |  |  | 0.41 |  | | | |  | - | 0.01 | |  | - | 3.19 | |  |  | 3.17 | |  | - | 0.01 | |  |  | 0.09 | |  | | |  |  | 0.54 | |  | - | 0.60 | |  |  | 1.69 |  |  | 0.93 |  |  | 0.35 |  |
| trial |  |  | 0.03 |  | - | 0.08 | |  |  | 0.14 | |  |  | 0.57 | |  |  | 0.57 |  | | | |  | - | 0.14 | |  | - | 0.46 | |  |  | 0.19 | |  | - | 0.82 | |  |  | 0.41 | |  | | |  |  | 0.05 | |  | - | 0.06 | |  |  | 0.16 |  |  | 0.89 |  |  | 0.37 |  |
| sex [male] |  | - | 0.06 |  | - | 0.74 | |  |  | 0.62 | |  | - | 0.17 | |  |  | 0.86 |  | | | |  |  | ‒ | |  |  | ‒ | |  |  | ‒ | |  |  | ‒ | |  |  | ‒ | |  | | |  |  | ‒ | |  |  | ‒ | |  |  | ‒ |  |  | ‒ |  |  | ‒ |  |
| rank [low] |  | **-** | **0.90** |  | **-** | **1.67** | |  | **-** | **0.13** | |  | **-** | **2.28** | |  |  | **0.02** |  | | | |  | - | 0.36 | |  | - | 2.83 | |  |  | 4.87 | |  |  | 2.10 | |  |  | 0.77 | |  | | |  | **-** | **0.92** | |  | **-** | **1.77** | |  | **-** | **0.07** |  | **-** | **2.13** |  |  | **0.03** |  |
| rank [mid] |  | - | 0.47 |  | - | 1.06 | |  |  | 0.11 | |  | - | 1.58 | |  |  | 0.11 |  | | | |  |  | 0.42 | |  | - | 1.28 | |  |  | 2.32 | |  |  | 2.11 | |  |  | 0.63 | |  | | |  | - | 0.59 | |  | - | 1.21 | |  |  | 0.04 |  | - | 1.83 |  |  | 0.07 |  |
| age category [old] |  |  | 0.12 |  | - | 0.43 | |  |  | 0.68 | |  |  | 0.44 | |  |  | 0.66 |  | | | |  |  | 0.24 | |  | - | 1.91 | |  |  | ‒ | |  |  | 2.40 | |  |  | 0.83 | |  | | |  |  | 0.09 | |  | - | 0.53 | |  |  | 0.71 |  |  | 0.28 |  |  | 0.78 |  |
| age category [very old] |  | - | 0.31 |  | - | 1.10 | |  |  | 0.49 | |  | - | 0.75 | |  |  | 0.45 |  | | | |  |  | ‒ | |  |  | ‒ | |  |  | ‒ | |  |  | ‒ | |  |  | ‒ | |  | | |  | - | 0.32 | |  | - | 1.14 | |  |  | 0.51 |  | - | 0.75 |  |  | 0.45 |  |
| degree |  |  | 0.11 |  | - | 1.09 | |  |  | 1.32 | |  |  | 0.19 | |  |  | 0.85 |  | | | |  |  | ‒ | |  |  | ‒ | |  |  | ‒ | |  |  | ‒ | |  |  | ‒ | |  | | |  | - | 0.15 | |  | - | 1.52 | |  |  | 1.21 |  | - | 0.22 |  |  | 0.82 |  |
| eigenvector |  | - | 0.77 |  | - | 2.13 | |  |  | 0.60 | |  | - | 1.10 | |  |  | 0.27 |  | | | |  | - | 0.81 | |  | - | 3.79 | |  |  | 3.38 | |  |  | 2.17 | |  |  | 0.60 | |  | | |  | - | 0.71 | |  | - | 2.18 | |  |  | 0.76 |  | - | 0.95 |  |  | 0.34 |  |
| betweenness |  |  | 0.69 |  | - | 0.76 | |  |  | 2.14 | |  |  | 0.93 | |  |  | 0.35 |  | | | |  |  | ‒ | |  |  | ‒ | |  |  | ‒ | |  |  | ‒ | |  |  | ‒ | |  | | |  |  | 1.13 | |  | - | 0.69 | |  |  | 2.94 |  |  | 1.22 |  |  | 0.22 |  |
| closeness |  | - | 0.78 |  | - | 2.54 | |  |  | 0.98 | |  | - | 0.87 | |  |  | 0.38 |  | | | |  |  | 0.54 | |  | - | 4.59 | |  |  | ‒ | |  |  | 5.67 | |  |  | 0.84 | |  | | |  | - | 0.89 | |  | - | 2.70 | |  |  | 0.91 |  | - | 0.97 |  |  | 0.33 |  |
|  |  |  |  |  |  |  | |  |  |  | |  |  |  | |  |  |  |  | | | |  |  |  | |  |  |  | |  |  |  | |  |  |  | |  |  |  | |  | | |  |  |  | |  |  |  | |  |  |  |  |  |  |  |  |  |  |
| *Transposition* | **Mixed-network model** | | | | | | | | | | | | | | | | | | | |  | **Male-network model** | | | | | | | | | | | | | | | | | | | |  | | | **Female-network model** | | | | | | | | | | | | | | | | | | |
| Parameter | Coefficient | | | CI_low | | | CI_high | | | | z value | | | | Pr (>\|z\|) | | | | |  | | Coefficient | | | | CI_low | | | | CI_high | | | | z value | | | | Pr (>\|z\|) | | | |  | | Coefficient | | | | | CI_low | | | | CI_high | | | | z value | | | Pr (>\|z\|) | | |  |
| (Intercept) |  | - | 0.88 |  | - | 2.11 | |  |  | 0.35 | |  | - | 1.40 | |  |  | 0.16 |  | | | |  | - | 1.84 | |  | - | 5.54 | |  |  | 1.87 | |  | - | 0.97 | |  |  | 0.33 | |  | | |  | - | 0.65 | |  | **-** | 1.82 | |  |  | 0.53 |  | **-** | 1.08 |  |  | 0.28 |  |
| trial |  |  | 0.06 |  | - | 0.05 | |  |  | 0.17 | |  |  | 1.05 | |  |  | 0.29 |  | | | |  | - | 0.21 | |  | - | 0.59 | |  |  | 0.16 | |  | - | 1.11 | |  |  | 0.27 | |  | | |  |  | 0.09 | |  | - | 0.03 | |  |  | 0.20 |  |  | 1.45 |  |  | 0.15 |  |
| sex [male] |  | - | 0.51 |  | - | 1.25 | |  |  | 0.23 | |  | - | 1.34 | |  |  | 0.18 |  | | | |  |  | ‒ | |  |  | ‒ | |  |  | ‒ | |  |  | ‒ | |  |  | ‒ | |  | | |  |  | ‒ | |  |  | ‒ | |  |  | ‒ |  |  | ‒ |  |  | ‒ |  |
| rank [low] |  | - | 0.27 |  | - | 1.05 | |  |  | 0.52 | |  | - | 0.66 | |  |  | 0.51 |  | | | |  |  | 1.78 | |  | - | 1.29 | |  |  | 4.84 | |  |  | 1.14 | |  |  | 0.26 | |  | | |  | - | 0.47 | |  | - | 1.33 | |  |  | 0.38 |  | - | 1.09 |  |  | 0.28 |  |
| rank [mid] |  | - | 0.15 |  | - | 0.75 | |  |  | 0.45 | |  | - | 0.48 | |  |  | 0.63 |  | | | |  |  | 1.53 | |  | - | 0.59 | |  |  | 3.65 | |  | - | 1.41 | |  |  | 0.16 | |  | | |  | - | 0.30 | |  | - | 0.94 | |  |  | 0.34 |  | - | 0.92 |  |  | 0.36 |  |
| age category [old] |  | - | 0.09 |  | - | 0.68 | |  |  | 0.49 | |  | - | 0.31 | |  |  | 0.75 |  | | | |  |  | 1.58 | |  | - | 1.06 | |  |  | 4.22 | |  |  | 1.17 | |  |  | 0.24 | |  | | |  | - | 0.10 | |  | - | 0.75 | |  |  | 0.54 |  | - | 0.31 |  |  | 0.75 |  |
| age category [very old] |  |  | 0.14 |  | - | 0.65 | |  |  | 0.93 | |  |  | 0.34 | |  |  | 0.73 |  | | | |  |  | ‒ | |  |  | ‒ | |  |  | ‒ | |  |  | ‒ | |  |  | ‒ | |  | | |  |  | 0.22 | |  | - | 0.60 | |  |  | 1.03 |  |  | 0.52 |  |  | 0.60 |  |
| degree |  |  | 0.07 |  | - | 1.18 | |  |  | 1.31 | |  |  | 0.11 | |  |  | 0.92 |  | | | |  |  | ‒ | |  |  | ‒ | |  |  | ‒ | |  |  | ‒ | |  |  | ‒ | |  | | |  | - | 0.26 | |  | - | 1.65 | |  |  | 1.13 |  | - | 0.37 |  |  | 0.71 |  |
| eigenvector |  | - | 0.94 |  | - | 2.36 | |  |  | 0.48 | |  | - | 1.29 | |  |  | 0.20 |  | | | |  | - | 0.46 | |  | **-** | 3.57 | |  |  | 2.65 | |  | - | 0.29 | |  |  | 0.77 | |  | | |  | - | 1.13 | |  | - | 2.67 | |  |  | 0.41 |  | - | 1.44 |  |  | 0.15 |  |
| betweenness |  | - | 0.07 |  | - | 1.53 | |  |  | 1.40 | |  | - | 0.09 | |  |  | 0.93 |  | | | |  |  | ‒ | |  |  | ‒ | |  |  | ‒ | |  |  | ‒ | |  |  | ‒ | |  | | |  | - | 0.22 | |  | - | 2.05 | |  |  | 1.61 |  | - | 0.23 |  |  | 0.82 |  |
| closeness |  |  | 0.94 |  | - | 0.86 | |  |  | 2.73 | |  |  | 1.02 | |  |  | 0.31 |  | | | |  | - | 0.93 | |  | - | 6.82 | |  |  | 4.97 | |  | - | 0.31 | |  |  | 0.76 | |  | | |  |  | 1.03 | |  | - | 0.80 | |  |  | 2.87 |  |  | 1.10 |  |  | 0.27 |  |
|  |  | | | | | | | | | | | | | | | | | | | |  |  | | | | | | | | | | | | | | | | | | | |  | | |  | | | | | | | | | | | | | | | | | | |
| *Relative number* | **Mixed-network model** | | | | | | | | | | | | | | | | | | | |  | **Male-network model** | | | | | | | | | | | | | | | | | | | |  | | | **Female-network model** | | | | | | | | | | | | | | | | | | |
| Parameter | Coefficient | | | CI_low | | | CI_high | | | | z value | | | | Pr (>\|z\|) | | | | |  | | Coefficient | | | | CI_low | | | | CI_high | | | | z value | | | | Pr (>\|z\|) | | | |  | | Coefficient | | | | | CI_low | | | | CI_high | | | | z value | | | Pr (>\|z\|) | | |  |
| (Intercept) |  |  | 0.76 |  | - | 0.03 | |  |  | 1.55 | |  |  | 1.89 | |  |  | 0.06 |  | | | |  |  | **2.92** | |  |  | **0.78** | |  |  | **5.07** | |  |  | **2.67** | |  |  | **0.01** | |  | | |  |  | 0.71 | |  | - | 0.05 | |  |  | 1.47 |  | - | 1.83 |  |  | 0.07 |  |
| trial |  | - | 0.01 |  | - | 0.03 | |  |  | 0.02 | |  | - | 0.41 | |  |  | 0.68 |  | | | |  |  | 0.00 | |  | - | 0.09 | |  |  | 0.08 | |  | - | 0.09 | |  |  | 0.93 | |  | | |  | - | 0.01 | |  |  | 0.04 | |  |  | 0.02 |  |  | -0.41 |  |  | 0.68 |  |
| sex [male] |  | - | 0.09 |  | - | 0.54 | |  |  | 0.36 | |  | - | 0.39 | |  |  | 0.70 |  | | | |  |  | ‒ | |  |  | ‒ | |  |  | ‒ | |  |  | ‒ | |  |  | ‒ | |  | | |  |  | ‒ | |  |  | ‒ | |  |  | ‒ |  |  | ‒ |  |  | 0.65 |  |
| rank [low] |  |  | 0.11 |  | - | 0.41 | |  |  | 0.63 | |  | - | 0.42 | |  |  | 0.67 |  | | | |  | - | 0.82 | |  | - | 2.50 | |  |  | 0.86 | |  | - | 0.96 | |  |  | 0.34 | |  | | |  |  | 0.13 | |  | - | 0.45 | |  |  | 0.71 |  |  | 0.45 |  |  | 0.87 |  |
| rank [mid] |  |  | 0.04 |  | - | 0.33 | |  |  | 0.42 | |  |  | 0.23 | |  |  | 0.82 |  | | | |  |  | 0.14 | |  | - | 0.94 | |  |  | 1.22 | |  |  | 0.25 | |  |  | 0.80 | |  | | |  |  | 0.03 | |  | - | 0.37 | |  |  | 0.43 |  |  | 0.17 |  |  | 0.98 |  |
| age category [old] |  | - | 0.03 |  | - | 0.38 | |  |  | 0.33 | |  | - | 0.15 | |  |  | 0.88 |  | | | |  | - | 0.57 | |  | - | 2.03 | |  |  | 0.88 | |  | - | 0.77 | |  |  | 0.44 | |  | | |  | - | 0.01 | |  | - | 0.41 | |  |  | 0.39 |  | - | 0.03 |  |  | 0.75 |  |
| age category [very old] |  |  | 0.07 |  | - | 0.43 | |  |  | 0.57 | |  | - | 0.27 | |  |  | 0.78 |  | | | |  |  | ‒ | |  |  | ‒ | |  |  | ‒ | |  |  | ‒ | |  |  | ‒ | |  | | |  |  | 0.09 | |  | - | 0.43 | |  |  | 0.61 |  |  | 0.32 |  |  | 0.41 |  |
| degree |  | - | 0.55 |  | - | 1.34 | |  |  | 0.24 | |  | - | 1.36 | |  |  | 0.17 |  | | | |  |  | ‒ | |  |  | ‒ | |  |  | ‒ | |  |  | ‒ | |  |  | ‒ | |  | | |  | - | 0.37 | |  | - | 1.25 | |  |  | 0.52 |  | - | 0.82 |  |  | 0.63 |  |
| eigenvector |  |  | 0.36 |  | - | 0.52 | |  |  | 1.24 | |  |  | 0.80 | |  |  | 0.42 |  | | | |  |  | 0.87 | |  | - | 1.02 | |  |  | 2.77 | |  |  | 0.90 | |  |  | 0.37 | |  | | |  |  | 0.23 | |  | - | 0.71 | |  |  | 1.17 |  |  | 0.49 |  |  | 0.48 |  |
| betweenness |  | - | 0.37 |  | - | 1.30 | |  |  | 0.57 | |  | - | 0.77 | |  |  | 0.44 |  | | | |  |  | ‒ | |  |  | ‒ | |  |  | ‒ | |  |  | ‒ | |  |  | ‒ | |  | | |  | - | 0.42 | |  | - | 1.58 | |  |  | 0.75 |  | - | 0.70 |  |  | 0.89 |  |
| closeness |  | - | 0.05 |  | - | 1.22 | |  |  | 1.11 | |  | - | 0.09 | |  |  | 0.93 |  | | | |  | **-** | **4.34** | |  | **-** | **7.64** | |  | **-** | **1.05** | |  | **-** | **2.58** | |  |  | **0.01** | |  | | |  | - | 0.08 | |  | - | 1.28 | |  |  | 1.12 |  | - | 0.13 |  |  | 0.07 |  |
|  |  |  |  |  |  |  | |  |  |  | |  |  |  | |  |  |  |  | | | |  |  |  | |  |  |  | |  |  |  | |  |  |  | |  |  |  | |  | | |  |  |  | |  |  |  | |  |  |  |  |  |  |  |  |  |  |
| *Noise* | **Mixed-network model** | | | | | | | | | | | | | | | | | | | |  | **Male-network model** | | | | | | | | | | | | | | | | | | | |  | | | **Female-network model** | | | | | | | | | | | | | | | | | | |
| Parameter | Coefficient | | | CI_low | | | CI_high | | | | z value | | | | Pr (>\|z\|) | | | | |  | | Coefficient | | | | CI_low | | | | CI_high | | | | z value | | | | Pr (>\|z\|) | | | |  | | Coefficient | | | | | CI_low | | | | CI_high | | | | z value | | | Pr (>\|z\|) | | |  |
| (Intercept) |  |  | 0.66 |  | - | 0.21 | |  |  | 1.52 | |  |  | 1.48 | |  |  | 0.14 |  | | | |  | - | 0.10 | |  | - | 2.30 | |  |  | 2.11 | |  | - | 0.08 | |  |  | 0.93 | |  | | |  |  | 0.63 | |  | **-** | 0.20 | |  |  | 1.45 |  |  | 1.48 |  |  | 0.14 |  |
| trial |  | - | 0.01 |  | - | 0.05 | |  |  | 0.03 | |  | - | 0.56 | |  |  | 0.57 |  | | | |  | - | 0.02 | |  | - | 0.13 | |  |  | 0.09 | |  | - | 0.31 | |  |  | 0.75 | |  | | |  | - | 0.01 | |  | - | 0.05 | |  |  | 0.03 |  | - | 0.49 |  |  | 0.63 |  |
| sex [male] |  | - | 0.23 |  | - | 0.73 | |  |  | 0.26 | |  | - | 0.91 | |  |  | 0.36 |  | | | |  |  | ‒ | |  |  | ‒ | |  |  | ‒ | |  |  | ‒ | |  |  | ‒ | |  | | |  |  | ‒ | |  |  | ‒ | |  |  | ‒ |  |  | ‒ |  |  | ‒ |  |
| rank [low] |  |  | 0.15 |  | - | 0.41 | |  |  | 0.72 | |  |  | 0.53 | |  |  | 0.60 |  | | | |  |  | 0.45 | |  | - | 1.31 | |  |  | 2.20 | |  |  | 0.50 | |  |  | 0.62 | |  | | |  |  | 0.20 | |  | - | 0.43 | |  |  | 0.84 |  |  | 0.63 |  |  | 0.53 |  |
| rank [mid] |  | - | 0.11 |  | - | 0.52 | |  |  | 0.31 | |  | - | 0.51 | |  |  | 0.61 |  | | | |  | - | 0.18 | |  | - | 1.07 | |  |  | 1.42 | |  |  | 0.28 | |  |  | 0.78 | |  | | |  | - | 0.12 | |  | - | 0.56 | |  |  | 0.32 |  | - | 0.52 |  |  | 0.60 |  |
| age category [old] |  | - | 0.35 |  | - | 0.76 | |  |  | 0.05 | |  | - | 1.70 | |  |  | 0.09 |  | | | |  |  | 0.20 | |  | - | 1.33 | |  |  | 1.73 | |  |  | 0.26 | |  |  | 0.79 | |  | | |  | - | 0.40 | |  | - | 0.86 | |  |  | 0.07 |  | - | 1.66 |  |  | 0.10 |  |
| age category [very old] |  |  | 0.27 |  | - | 0.29 | |  |  | 0.83 | |  |  | 0.94 | |  |  | 0.35 |  | | | |  |  | ‒ | |  |  | ‒ | |  |  | ‒ | |  |  | ‒ | |  |  | ‒ | |  | | |  |  | 0.28 | |  | - | 0.30 | |  |  | 0.86 |  |  | 0.95 |  |  | 0.34 |  |
| degree |  | - | 0.24 |  | - | 1.10 | |  |  | 0.62 | |  | - | 0.54 | |  |  | 0.59 |  | | | |  |  | ‒ | |  |  | ‒ | |  |  | ‒ | |  |  | ‒ | |  |  | ‒ | |  | | |  | - | 0.38 | |  | - | 1.34 | |  |  | 0.59 |  | - | 0.77 |  |  | 0.44 |  |
| eigenvector |  |  | 0.12 |  | - | 0.86 | |  |  | 1.10 | |  |  | 0.24 | |  |  | 0.81 |  | | | |  | **-** | **1.03** | |  | - | 3.19 | |  |  | 1.13 | |  | - | 0.93 | |  |  | 0.35 | |  | | |  |  | 0.26 | |  | - | 0.79 | |  |  | 1.30 |  |  | 0.48 |  |  | 0.63 |  |
| betweenness |  |  | 0.65 |  | - | 0.40 | |  |  | 1.70 | |  |  | 1.21 | |  |  | 0.22 |  | | | |  |  | ‒ | |  |  | ‒ | |  |  | ‒ | |  |  | ‒ | |  |  | ‒ | |  | | |  | - | 0.60 | |  | - | 0.71 | |  |  | 1.90 |  |  | 0.89 |  |  | 0.37 |  |
| closeness |  | - | 0.83 |  | - | 2.11 | |  |  | 0.45 | |  | - | 1.28 | |  |  | 0.20 |  | | | |  | - | 0.07 | |  | - | 3.66 | |  |  | 3.52 | |  | - | 0.04 | |  |  | 0.97 | |  | | |  | - | 0.85 | |  | - | 2.16 | |  |  | 0.47 |  | - | 1.26 |  |  | 0.21 |  |

All models were built using binomial generalized linear mixed models with a log link function and included random effect of Japanese macaque identity (ID). The mixed-network model included social centrality variables calculated within a network that included both males and females. The male-network model derived centrality variables exclusively from male-male social network, and the female-network model did so from female-female social network. Bold values indicate significant effects. For sex, rank, and age category, the reference categories were “female”, “high”, and “adult”, respectively. Note that none of the models explained the task performance better than the null models in LRTs (see Results).

Table S3. The results of GLMMs in the social domain.

| *Social domain* | **Mixed-network model** | | | | | | | | | | | | | | | | | | | |  | **Male-network model** | | | | | | | | | | | | | | | | | | | |  | | **Female-network model** | | | | | | | | | | | | | | | | | |
| --- | --- | --- | --- | --- | --- | --- | --- | --- | --- | --- | --- | --- | --- | --- | --- | --- | --- | --- | --- | --- | --- | --- | --- | --- | --- | --- | --- | --- | --- | --- | --- | --- | --- | --- | --- | --- | --- | --- | --- | --- | --- | --- | --- | --- | --- | --- | --- | --- | --- | --- | --- | --- | --- | --- | --- | --- | --- | --- | --- | --- | --- |
| Parameter | Coefficient | | | CI_low | | | CI_high | | | | z value | | | | Pr (>\|z\|) | | | | |  | | Coefficient | | | | CI_low | | | | CI_high | | | | z value | | | | Pr (>\|z\|) | | | |  | | Coefficient | | | | CI_low | | | | CI_high | | | | z value | | | Pr (>\|z\|) | | |
| (Intercept) |  |  | 0.11 |  | **-** | 0.50 | |  |  | 0.72 | |  |  | 0.36 | |  |  | 0.72 |  | | | |  | - | 0.39 | |  | - | 0.49 | |  |  | 1.26 | |  |  | 0.86 | |  |  | 0.39 | |  | |  | - | -0.23 | |  | - | 0.82 | |  |  | 0.36 |  | - | 0.78 |  |  | 0.44 |
| sex [male] |  | - | 0.08 |  | - | 0.42 | |  |  | 0.26 | |  | - | 0.45 | |  |  | 0.65 |  | | | |  |  | ‒ | |  |  | ‒ | |  |  | ‒ | |  |  | ‒ | |  |  | ‒ | |  | |  |  | ‒ | |  |  | ‒ | |  |  | ‒ |  |  | ‒ |  |  | ‒ |
| rank [low] |  |  | 0.26 |  | - | 0.14 | |  |  | 0.66 | |  |  | 1.29 | |  |  | 0.20 |  | | | |  | - | 0.30 | |  | - | 1.08 | |  |  | 0.48 | |  | - | 0.75 | |  |  | 0.45 | |  | |  |  | 0.42 | |  | - | 0.02 | |  |  | 0.86 |  |  | 1.86 |  |  | 0.06 |
| rank [mid] |  |  | 0.19 |  | - | 0.12 | |  |  | 0.50 | |  |  | 1.21 | |  |  | 0.23 |  | | | |  |  | 0.38 | |  | - | 0.40 | |  |  | 1.15 | |  |  | 0.94 | |  |  | 0.35 | |  | |  |  | 0.23 | |  | - | 0.10 | |  |  | 0.56 |  |  | 1.36 |  |  | 0.17 |
| age category [old] |  |  | 0.09 |  | - | 0.21 | |  |  | 0.38 | |  |  | 0.59 | |  |  | 0.56 |  | | | |  |  | ‒ | |  |  | ‒ | |  |  | ‒ | |  |  | ‒ | |  |  | ‒ | |  | |  | - | 0.10 | |  | - | 0.23 | |  |  | 0.43 |  |  | 0.59 |  |  | 0.56 |
| age category [very old] |  | - | 0.15 |  | - | 0.53 | |  |  | 0.24 | |  | - | 0.74 | |  |  | 0.46 |  | | | |  |  | ‒ | |  |  | ‒ | |  |  | ‒ | |  |  | ‒ | |  |  | ‒ | |  | |  | - | 0.23 | |  | - | 0.65 | |  |  | 0.20 |  | - | 1.03 |  |  | 0.30 |
| task [social cue] |  | - | 0.01 |  | - | 0.21 | |  |  | 0.18 | |  | - | 0.12 | |  |  | 0.90 |  | | | |  | - | 0.22 | |  | - | 0.84 | |  |  | 0.40 | |  | - | 0.70 | |  |  | 0.49 | |  | |  |  | 0.04 | |  | - | 0.17 | |  |  | 0.25 |  |  | 0.39 |  |  | 0.69 |
| degree |  |  | 0.53 |  | - | 0.08 | |  |  | 1.14 | |  |  | 1.69 | |  |  | 0.09 |  | | | |  |  | ‒ | |  |  | ‒ | |  |  | ‒ | |  |  | ‒ | |  |  | ‒ | |  | |  |  | 0.71 | |  | - | 0.03 | |  |  | 1.39 |  |  | 2.04 |  |  | 0.04 |
| eigenvector |  |  | 0.15 |  | - | 0.58 | |  |  | 0.88 | |  |  | 0.40 | |  |  | 0.69 |  | | | |  | - | 1.20 | |  | - | 2.67 | |  |  | 0.26 | |  | - | 1.61 | |  |  | 0.11 | |  | |  | - | 0.03 | |  | - | 0.80 | |  |  | 0.74 |  | - | 0.08 |  |  | 0.94 |
| betweenness |  |  | 0.27 |  | - | 0.53 | |  |  | 1.07 | |  |  | 0.66 | |  |  | 0.51 |  | | | |  |  | ‒ | |  |  | ‒ | |  |  | ‒ | |  |  | ‒ | |  |  | ‒ | |  | |  |  | 0.11 | |  | - | 0.90 | |  |  | 1.11 |  |  | 0.21 |  |  | 0.83 |
| closeness |  | - | 0.53 |  | - | 1.46 | |  |  | 0.40 | |  | - | 1.12 | |  |  | 0.26 |  | | | |  |  | 0.43 | |  | - | 1.80 | |  |  | 2.65 | |  |  | 0.38 | |  |  | 0.71 | |  | |  | - | 0.02 | |  | - | 0.99 | |  |  | 0.95 |  | - | 0.04 |  |  | 0.97 |

All models were built using binomial generalized linear mixed models with a log link function and included random effect of Japanese macaque identity (ID). The mixed-network model included social centrality variables calculated within a network that included both males and females. The male-network model derived centrality variables exclusively from male-male social network, and the female-network model did so from female-female social network. Bold values indicate significant effects. For sex, rank, and age category, the reference categories were “female”, “high”, and “adult”, respectively. Note that none of the models explained the task performance better than the null models in LRTs (see Results).

Table S4. The results of GLMMs in the social cue and gaze perception task.

| *Social cue* | **Mixed-network model** | | | | | | | | | | | | | | | | | | | |  | **Male-network model** | | | | | | | | | | | | | | | | | | | |  | | **Female-network model** | | | | | | | | | | | | | | | | | | |  |
| --- | --- | --- | --- | --- | --- | --- | --- | --- | --- | --- | --- | --- | --- | --- | --- | --- | --- | --- | --- | --- | --- | --- | --- | --- | --- | --- | --- | --- | --- | --- | --- | --- | --- | --- | --- | --- | --- | --- | --- | --- | --- | --- | --- | --- | --- | --- | --- | --- | --- | --- | --- | --- | --- | --- | --- | --- | --- | --- | --- | --- | --- | --- | --- |
| Parameter | Coefficient | | | CI_low | | | CI_high | | | | z value | | | | Pr (>\|z\|) | | | | |  | | Coefficient | | | | CI_low | | | | CI_high | | | | z value | | | | Pr (>\|z\|) | | | |  | | Coefficient | | | | | CI_low | | | | CI_high | | | | z value | | | Pr (>\|z\|) | | |  |
| (Intercept) |  |  | 0.00 |  | - | 0.83 | |  |  | 0.83 | |  |  | 0.00 | |  |  | 1.00 |  | | | |  |  | 1.01 | |  | - | 1.26 | |  |  | 3.27 | |  |  | 0.87 | |  |  | 0.38 | |  | | |  | **-** | 0.19 | |  | **-** | 0.99 | |  |  | 0.61 |  |  | -0.46 |  |  | 0.64 |  |
| trial |  |  | 0.01 |  | - | 0.02 | |  |  | 0.05 | |  |  | 0.66 | |  |  | 0.51 |  | | | |  |  | 0.03 | |  | - | 0.09 | |  |  | 0.14 | |  |  | 0.46 | |  |  | 0.65 | |  | | |  |  | 0.01 | |  | - | 0.03 | |  |  | 0.05 |  |  | 0.54 |  |  | 0.59 |  |
| sex [male] |  | - | 0.29 |  | - | 0.77 | |  |  | 0.19 | |  | - | 1.20 | |  |  | 0.23 |  | | | |  |  | ‒ | |  |  | ‒ | |  |  | ‒ | |  |  | ‒ | |  |  | ‒ | |  | | |  |  | ‒ | |  |  | ‒ | |  |  | ‒ |  |  | ‒ |  |  | ‒ |  |
| rank [low] |  |  | 0.39 |  | - | 0.15 | |  |  | 0.93 | |  |  | 1.41 | |  |  | 0.16 |  | | | |  | - | 0.99 | |  | - | 2.78 | |  |  | 0.80 | |  | - | 1.09 | |  |  | 0.28 | |  | | |  |  | 0.58 | |  | - | 0.02 | |  |  | 1.19 |  |  | 1.89 |  |  | 0.06 |  |
| rank [mid] |  |  | 0.16 |  | - | 0.26 | |  |  | 0.57 | |  |  | 0.75 | |  |  | 0.46 |  | | | |  |  | 0.29 | |  | - | 0.93 | |  |  | 1.51 | |  |  | 0.47 | |  |  | 0.64 | |  | | |  |  | 0.17 | |  | - | 0.27 | |  |  | 0.61 |  |  | 0.75 |  |  | 0.45 |  |
| age category [old] |  | - | 0.15 |  | - | 0.25 | |  |  | 0.56 | |  |  | 0.74 | |  |  | 0.46 |  | | | |  |  | -0.15 | |  | - | 1.73 | |  |  | 1.43 | |  | - | 0.18 | |  |  | 0.85 | |  | | |  |  | 0.14 | |  | - | 0.31 | |  |  | 0.60 |  |  | 0.62 |  |  | 0.54 |  |
| age category [very old] |  | **-** | 0.22 |  | **-** | 0.77 | |  | **-** | 0.33 | |  | **-** | 0.80 | |  |  | 0.43 |  | | | |  |  | ‒ | |  |  | ‒ | |  |  | ‒ | |  |  | ‒ | |  |  | ‒ | |  | | |  | - | 0.29 | |  | - | 0.86 | |  |  | 0.28 |  | - | 0.99 |  |  | 0.32 |  |
| degree |  |  | 0.78 |  | - | 0.08 | |  |  | 1.64 | |  |  | 1.79 | |  |  | 0.07 |  | | | |  |  | ‒ | |  |  | ‒ | |  |  | ‒ | |  |  | ‒ | |  |  | ‒ | |  | | |  |  | 0.78 | |  | - | 0.18 | |  |  | 1.75 |  |  | 1.59 |  |  | 0.11 |  |
| eigenvector |  |  | 0.01 |  | - | 0.95 | |  |  | 0.97 | |  |  | 0.02 | |  |  | 0.98 |  | | | |  | - | 1.18 | |  | - | 3.29 | |  |  | 0.93 | |  | - | 1.09 | |  |  | 0.27 | |  | | |  |  | 0.03 | |  | - | 1.00 | |  |  | 1.06 |  |  | 0.06 |  |  | 0.96 |  |
| betweenness |  |  | 0.63 |  | - | 0.41 | |  |  | 1.66 | |  |  | 1.18 | |  |  | 0.24 |  | | | |  |  | ‒ | |  |  | ‒ | |  |  | ‒ | |  |  | ‒ | |  |  | ‒ | |  | | |  |  | 0.75 | |  | - | 0.58 | |  |  | 2.07 |  |  | 1.10 |  |  | 0.27 |  |
| closeness |  | - | 0.68 |  | - | 1.91 | |  |  | 0.55 | |  | - | 1.09 | |  |  | 0.28 |  | | | |  | - | 0.90 | |  | - | 4.48 | |  |  | 2.68 | |  | - | 0.49 | |  |  | 0.62 | |  | | |  | - | 0.40 | |  | - | 1.67 | |  |  | 0.88 |  | - | 0.61 |  |  | 0.54 |  |
|  |  |  |  |  |  |  | |  |  |  | |  |  |  | |  |  |  |  | | | |  |  |  | |  |  |  | |  |  |  | |  |  |  | |  |  |  | |  | | |  |  |  | |  |  |  | |  |  |  |  |  |  |  |  |  |  |
| *Gaze perception* | **Mixed-network model** | | | | | | | | | | | | | | | | | | | |  | **Male-network model** | | | | | | | | | | | | | | | | | | | |  | | | **Female-network model** | | | | | | | | | | | | | | | | | | |
| Parameter | Coefficient | | | CI_low | | | CI_high | | | | z value | | | | Pr (>\|z\|) | | | | |  | | Coefficient | | | | CI_low | | | | CI_high | | | | z value | | | | Pr (>\|z\|) | | | |  | | Coefficient | | | | | CI_low | | | | CI_high | | | | z value | | | Pr (>\|z\|) | | |  |
| (Intercept) |  | - | 0.12 |  | - | 1.08 | |  |  | 0.85 | |  | - | 0.24 | |  |  | 0.81 |  | | | |  | - | 0.56 | |  | - | 3.02 | |  |  | 1.90 | |  | - | 0.45 | |  |  | 0.65 | |  | | |  | - | 0.61 | |  | - | 1.59 | |  |  | 0.38 |  | - | 1.20 |  |  | 0.23 |  |
| trial |  |  | 0.06 |  | - | 0.01 | |  |  | 0.12 | |  |  | 1.74 | |  |  | 0.08 |  | | | |  |  | 0.05 | |  | - | 0.12 | |  |  | 0.23 | |  |  | 0.59 | |  |  | 0.55 | |  | | |  |  | 0.06 | |  | - | 0.01 | |  |  | 0.13 |  |  | 1.65 |  |  | 0.10 |  |
| sex [male] |  | - | 0.10 |  | - | 0.40 | |  |  | 0.60 | |  |  | 0.37 | |  |  | 0.71 |  | | | |  |  | ‒ | |  |  | ‒ | |  |  | ‒ | |  |  | ‒ | |  |  | ‒ | |  | | |  |  | ‒ | |  |  | ‒ | |  |  | ‒ |  |  | ‒ |  |  | ‒ |  |
| rank [low] |  | - | 0.19 |  | - | 0.42 | |  |  | 0.79 | |  |  | 0.61 | |  |  | 0.54 |  | | | |  |  | 0.43 | |  |  | -1.99 | |  |  | 2.85 | |  |  | 0.35 | |  |  | 0.73 | |  | | |  |  | 0.32 | |  | - | 0.37 | |  |  | 1.01 |  |  | 0.91 |  |  | 0.36 |  |
| rank [mid] |  |  | 0.25 |  | - | 0.23 | |  |  | 0.72 | |  |  | 1.02 | |  |  | 0.31 |  | | | |  | - | 0.41 | |  | - | -1.78 | |  |  | 2.59 | |  |  | 0.35 | |  |  | 0.72 | |  | | |  |  | 0.32 | |  | - | 0.21 | |  |  | 0.84 |  |  | 1.18 |  |  | 0.24 |  |
| age category [old] |  |  | 0.08 |  | - | 0.36 | |  |  | 0.52 | |  |  | 0.36 | |  |  | 0.72 |  | | | |  |  | 0.58 | |  |  | -1.31 | |  |  | 2.47 | |  |  | 0.60 | |  |  | 0.55 | |  | | |  |  | 0.08 | |  | - | 0.44 | |  |  | 0.59 |  |  | 0.29 |  |  | 0.77 |  |
| age category [very old] |  | - | 0.07 |  | - | 0.64 | |  |  | 0.49 | |  | - | 0.25 | |  |  | 0.80 |  | | | |  |  | 0.01 | |  |  | -2.61 | |  |  | 2.62 | |  |  | 0.01 | |  |  | 0.99 | |  | | |  | - | 0.10 | |  |  | 0.80 | |  |  | 0.59 |  | - | 0.29 |  |  | 0.77 |  |
| degree |  |  | 0.31 |  | - | 0.58 | |  |  | 1.20 | |  |  | 0.68 | |  |  | 0.50 |  | | | |  |  | ‒ | |  |  | ‒ | |  |  | ‒ | |  |  | ‒ | |  |  | ‒ | |  | | |  |  | 0.64 | |  | - | 0.38 | |  |  | 1.66 |  |  | 1.22 |  |  | 0.22 |  |
| eigenvector |  |  | 0.28 |  | - | 0.85 | |  |  | 1.41 | |  |  | 0.48 | |  |  | 0.63 |  | | | |  | - | 1.25 | |  |  | -3.46 | |  |  | 0.96 | |  | - | 1.11 | |  |  | 0.27 | |  | | |  | - | 0.09 | |  | - | 1.31 | |  |  | 1.13 |  | - | 0.14 |  |  | 0.89 |  |
| betweenness |  | - | 0.24 |  | - | 1.53 | |  |  | 1.04 | |  | - | 0.37 | |  |  | 0.71 |  | | | |  |  | ‒ | |  |  | ‒ | |  |  | ‒ | |  |  | ‒ | |  |  | ‒ | |  | | |  | - | 0.80 | |  | - | 2.51 | |  |  | 0.91 |  | - | 0.92 |  |  | 0.36 |  |
| closeness |  | - | 0.40 |  | - | 1.84 | |  |  | 1.05 | |  | - | 0.54 | |  |  | 0.59 |  | | | |  |  | 0.91 | |  |  | -1.87 | |  |  | 3.69 | |  |  | 0.64 | |  |  | 0.52 | |  | | |  |  | 0.46 | |  | - | 1.14 | |  |  | 2.07 |  |  | 0.57 |  |  | 0.57 |  |

All models were built using binomial generalized linear mixed models with a log link function and included random effect of Japanese macaque identity (ID). The mixed-network model included social centrality variables calculated within a network that included both males and females. The male-network model derived centrality variables exclusively from male-male social network, and the female-network model did so from female-female social network. Bold values indicate significant effects. For sex, rank, and age category, the reference categories were “female”, “high”, and “adult”, respectively. Note that none of the models explained the task performance better than the null models in LRTs (see Results).

Table S5. The results of GLMMs in the inhibitory domain.

| *Inhibitory domain* | **Mixed-network model** | | | | | | | | | | | | | | | | | | | |  | **Male-network model** | | | | | | | | | | | | | | | | | | | |  | | **Female-network model** | | | | | | | | | | | | | | | | | |
| --- | --- | --- | --- | --- | --- | --- | --- | --- | --- | --- | --- | --- | --- | --- | --- | --- | --- | --- | --- | --- | --- | --- | --- | --- | --- | --- | --- | --- | --- | --- | --- | --- | --- | --- | --- | --- | --- | --- | --- | --- | --- | --- | --- | --- | --- | --- | --- | --- | --- | --- | --- | --- | --- | --- | --- | --- | --- | --- | --- | --- | --- |
| Parameter | Coefficient | | | CI_low | | | CI_high | | | | z value | | | | Pr (>\|z\|) | | | | |  | | Coefficient | | | | CI_low | | | | CI_high | | | | z value | | | | Pr (>\|z\|) | | | |  | | Coefficient | | | | CI_low | | | | CI_high | | | | z value | | | Pr (>\|z\|) | | |
| (Intercept) |  | **-** | **1.43** |  | **-** | **2.83** | |  | **-** | **0.03** | |  | **-** | **2.00** | |  |  | **0.04** |  | | | |  | **-** | **1.50** | |  | **-** | **2.77** | |  | **-** | **0.24** | |  | **-** | **2.34** | |  |  | **0.02** | |  | |  | **-** | **1.83** | |  | **-** | **3.19** | |  | **-** | **0.48** |  | **-** | **2.65** |  |  | **0.01** |
| sex [male] |  | - | 0.16 |  | - | 0.85 | |  |  | 0.53 | |  | - | 0.45 | |  |  | 0.65 |  | | | |  |  | ‒ | |  |  | ‒ | |  |  | ‒ | |  |  | ‒ | |  |  | ‒ | |  | |  |  | ‒ | |  |  | ‒ | |  |  | ‒ |  |  | ‒ |  |  | ‒ |
| rank [low] |  |  | 0.10 |  | - | 0.79 | |  |  | 1.00 | |  |  | 0.23 | |  |  | 0.82 |  | | | |  |  | 0.79 | |  | - | 0.26 | |  |  | 1.85 | |  |  | 1.47 | |  |  | 0.14 | |  | |  |  | 0.01 | |  | - | 0.97 | |  |  | 1.00 |  |  | 0.03 |  |  | 0.98 |
| rank [mid] |  |  | 0.53 |  | - | 0.22 | |  |  | 1.27 | |  |  | 1.39 | |  |  | 0.16 |  | | | |  | - | 0.47 | |  | - | 1.46 | |  |  | 0.52 | |  | - | 0.93 | |  |  | 0.35 | |  | |  |  | 0.63 | |  | - | 0.18 | |  |  | 1.44 |  |  | 1.52 |  |  | 0.13 |
| age category [old] |  | - | 0.22 |  | - | 0.83 | |  |  | 0.40 | |  | - | 0.69 | |  |  | 0.49 |  | | | |  |  | ‒ | |  |  | ‒ | |  |  | ‒ | |  |  | ‒ | |  |  | ‒ | |  | |  | - | 0.26 | |  | - | 0.97 | |  |  | 0.45 |  | - | 0.71 |  |  | 0.48 |
| age category [very old] |  | - | 0.74 |  | - | 1.54 | |  |  | 0.05 | |  | - | 1.84 | |  |  | 0.07 |  | | | |  |  | ‒ | |  |  | ‒ | |  |  | ‒ | |  |  | ‒ | |  |  | ‒ | |  | |  | - | 0.58 | |  | - | 1.48 | |  |  | 0.31 |  | - | 1.27 |  |  | 0.20 |
| task [cylinder] |  |  | **1.01** |  |  | **0.74** | |  |  | **1.27** | |  |  | **7.45** | |  |  | **0.00** |  | | | |  |  | 0.55 | |  | - | 0.12 | |  |  | 1.25 | |  |  | 1.61 | |  |  | 0.11 | |  | |  |  | **1.09** | |  |  | **0.80** | |  |  | **1.37** |  |  | **7.37** |  |  | **0.00** |
| degree |  | - | 0.52 |  | - | 1.87 | |  |  | 0.82 | |  | - | 0.76 | |  |  | 0.45 |  | | | |  |  | ‒ | |  |  | ‒ | |  |  | ‒ | |  |  | ‒ | |  |  | ‒ | |  | |  | - | 0.37 | |  | - | 1.90 | |  |  | 1.16 |  | - | 0.48 |  |  | 0.63 |
| eigenvector |  |  | **2.96** |  |  | **1.12** | |  |  | **4.79** | |  |  | **3.16** | |  |  | **0.00** |  | | | |  |  | **2.11** | |  |  | **0.12** | |  |  | **4.10** | |  |  | **2.08** | |  |  | **0.04** | |  | |  |  | **2.78** | |  |  | **0.82** | |  |  | **4.73** |  |  | **2.79** |  |  | **0.01** |
| betweenness |  | - | 0.75 |  | - | 2.59 | |  |  | 1.10 | |  | - | 0.80 | |  |  | 0.43 |  | | | |  |  | ‒ | |  |  | ‒ | |  |  | ‒ | |  |  | ‒ | |  |  | ‒ | |  | |  | - | 1.32 | |  | - | 3.66 | |  |  | 1.02 |  | - | 1.11 |  |  | 0.27 |
| closeness |  |  | 0.13 |  | - | 1.95 | |  |  | 2.21 | |  |  | 0.12 | |  |  | 0.90 |  | | | |  |  | 0.39 | |  | - | 2.04 | |  |  | 2.83 | |  |  | 0.32 | |  |  | 0.75 | |  | |  |  | 0.85 | |  | - | 1.31 | |  |  | 3.01 |  |  | 0.77 |  |  | 0.44 |

All models were built using binomial generalized linear mixed models with a log link function and included random effect of Japanese macaque identity (ID). The mixed-network model included social centrality variables calculated within a network that included both males and females. The male-network model derived centrality variables exclusively from male-male social network, and the female-network model did so from female-female social network. Bold values indicate significant effects. For sex, rank, and age category, the reference categories were “female”, “high”, and “adult”, respectively.

Table S6. The results of GLMMs in the A-not-B and Cylinder task.

| *A-not-B* | **Mixed-network model** | | | | | | | | | | | | | | | | | | | |  | **Male-network model** | | | | | | | | | | | | | | | | | | | |  | | **Female-network model** | | | | | | | | | | | | | | | | | | |  |
| --- | --- | --- | --- | --- | --- | --- | --- | --- | --- | --- | --- | --- | --- | --- | --- | --- | --- | --- | --- | --- | --- | --- | --- | --- | --- | --- | --- | --- | --- | --- | --- | --- | --- | --- | --- | --- | --- | --- | --- | --- | --- | --- | --- | --- | --- | --- | --- | --- | --- | --- | --- | --- | --- | --- | --- | --- | --- | --- | --- | --- | --- | --- | --- |
| Parameter | Coefficient | | | CI_low | | | CI_high | | | | z value | | | | Pr (>\|z\|) | | | | |  | | Coefficient | | | | CI_low | | | | CI_high | | | | z value | | | | Pr (>\|z\|) | | | |  | | Coefficient | | | | | CI_low | | | | CI_high | | | | z value | | | Pr (>\|z\|) | | |  |
| (Intercept) |  | **-** | **2.84** |  |  | **5.66** | |  | **-** | **0.02** | |  | **-** | **1.98** | |  |  | **0.05** |  | | | |  | **-** | **3.54** | |  | **-** | **6.54** | |  | **-** | **0.54** | |  | **-** | **2.32** | |  |  | **0.02** | |  | | |  | **-** | **3.40** | |  | **-** | **6.18** | |  | **-** | **0.62** |  | **-** | **2.40** |  |  | **0.02** |  |
| trial |  |  | **0.43** |  |  | **0.24** | |  |  | **0.62** | |  |  | **4.46** | |  |  | **0.00** |  | | | |  |  | **0.59** | |  |  | **0.07** | |  |  | **1.11** | |  |  | **2.24** | |  |  | **0.03** | |  | | |  |  | **0.41** | |  |  | **0.20** | |  |  | **0.61** |  |  | **3.88** |  |  | **0.00** |  |
| sex [male] |  |  | 0.65 |  | - | 0.85 | |  |  | 2.15 | |  |  | 0.85 | |  |  | 0.40 |  | | | |  |  | ‒ | |  |  | ‒ | |  |  | ‒ | |  |  | ‒ | |  |  | ‒ | |  | | |  |  | ‒ | |  |  | ‒ | |  |  | ‒ |  |  | ‒ |  |  | ‒ |  |
| rank [low] |  |  | 0.83 |  | - | 1.02 | |  |  | 2.68 | |  |  | 0.88 | |  |  | 0.38 |  | | | |  |  | 2.29 | |  | - | 0.28 | |  |  | 4.87 | |  |  | 1.74 | |  |  | 0.08 | |  | | |  |  | 0.36 | |  | - | 1.82 | |  |  | 2.53 |  |  | 0.32 |  |  | 0.75 |  |
| rank [mid] |  |  | 0.74 |  | - | 0.67 | |  |  | 2.15 | |  |  | 1.03 | |  |  | 0.30 |  | | | |  | - | 0.05 | |  | - | 2.42 | |  |  | 2.32 | |  | - | 0.04 | |  |  | 0.97 | |  | | |  |  | 0.89 | |  | - | 0.67 | |  |  | 2.45 |  |  | 1.12 |  |  | 0.26 |  |
| age category [old] |  | - | 0.77 |  | - | 2.09 | |  |  | 0.54 | |  | - | 1.15 | |  |  | 0.25 |  | | | |  |  | ‒ | |  |  | ‒ | |  |  | ‒ | |  |  | ‒ | |  |  | ‒ | |  | | |  | - | 0.82 | |  | - | 2.36 | |  |  | 0.71 |  | - | 1.05 |  |  | 0.29 |  |
| age category [very old] |  | **-** | **2.59** |  | **-** | **4.71** | |  | **-** | **0.47** | |  | **-** | **2.40** | |  |  | **0.02** |  | | | |  |  | ‒ | |  |  | ‒ | |  |  | ‒ | |  |  | ‒ | |  |  | ‒ | |  | | |  | - | 2.17 | |  | - | 4.51 | |  |  | 0.17 |  | - | 1.82 |  |  | 0.07 |  |
| degree |  |  | 2.19 |  | - | 0.63 | |  |  | 5.01 | |  | - | 1.52 | |  |  | 0.13 |  | | | |  |  | ‒ | |  |  | ‒ | |  |  | ‒ | |  |  | ‒ | |  |  | ‒ | |  | | |  |  | 2.64 | |  | - | 0.61 | |  |  | 5.90 |  |  | 1.59 |  |  | 0.11 |  |
| eigenvector |  |  | 3.13 |  | - | 0.24 | |  |  | 6.50 | |  |  | 1.82 | |  |  | 0.07 |  | | | |  | - | 0.03 | |  | - | 3.44 | |  |  | 3.38 | |  | - | 0.02 | |  |  | 0.99 | |  | | |  |  | 2.89 | |  | - | 0.79 | |  |  | 6.56 |  |  | 1.54 |  |  | 0.12 |  |
| betweenness |  | - | 2.66 |  | - | 6.39 | |  |  | 1.07 | |  | - | 1.40 | |  |  | 0.16 |  | | | |  |  | ‒ | |  |  | ‒ | |  |  | ‒ | |  |  | ‒ | |  |  | ‒ | |  | | |  | - | 3.05 | |  | - | 7.63 | |  |  | 1.53 |  | - | 1.30 |  |  | 0.19 |  |
| closeness |  | - | 2.14 |  | - | 6.44 | |  |  | 2.16 | |  | - | 0.98 | |  |  | 0.33 |  | | | |  |  | ‒ | |  |  | ‒ | |  |  | ‒ | |  |  | ‒ | |  |  | ‒ | |  | | |  | - | 1.36 | |  | - | 5.86 | |  |  | 3.15 |  | - | 0.59 |  |  | 0.56 |  |
|  |  |  |  |  |  |  | |  |  |  | |  |  |  | |  |  |  |  | | | |  |  |  | |  |  |  | |  |  |  | |  |  |  | |  |  |  | |  | | |  |  |  | |  |  |  | |  |  |  |  |  |  |  |  |  |  |
| *Cylinder* | **Mixed-network model** | | | | | | | | | | | | | | | | | | | |  | **Male-network model** | | | | | | | | | | | | | | | | | | | |  | | | **Female-network model** | | | | | | | | | | | | | | | | | | |
| Parameter | Coefficient | | | CI_low | | | CI_high | | | | z value | | | | Pr (>\|z\|) | | | | |  | | Coefficient | | | | CI_low | | | | CI_high | | | | z value | | | | Pr (>\|z\|) | | | |  | | Coefficient | | | | | CI_low | | | | CI_high | | | | z value | | | Pr (>\|z\|) | | |  |
| (Intercept) |  | - | 1.30 |  | - | 3.11 | |  |  | 0.52 | |  | - | 1.40 | |  |  | 0.16 |  | | | |  | - | 1.50 | |  | - | 3.36 | |  |  | 0.36 | |  |  | -1.58 | |  |  | 0.11 | |  | | |  | **-** | **1.73** | |  | **-** | **3.46** | |  | **-** | **0.01** |  | **-** | **1.97** |  |  | **0.05** |  |
| trial |  |  | **0.19** |  |  | **0.14** | |  |  | **0.24** | |  |  | **7.34** | |  |  | **0.00** |  | | | |  |  | **0.14** | |  |  | **0.00** | |  |  | **0.28** | |  |  | **2.02** | |  |  | **0.04** | |  | | |  |  | **0.19** | |  |  | **0.14** | |  |  | **0.25** |  |  | **7.06** |  |  | **0.00** |  |
| sex [male] |  | - | 0.36 |  | - | 1.26 | |  |  | 0.53 | |  | - | 0.79 | |  |  | 0.43 |  | | | |  |  | ‒ | |  |  | ‒ | |  |  | ‒ | |  |  | ‒ | |  |  | ‒ | |  | | |  |  | ‒ | |  |  | ‒ | |  |  | ‒ |  |  | ‒ |  |  | ‒ |  |
| rank [low] |  | - | 0.01 |  | - | 1.17 | |  |  | 1.14 | |  | - | 0.03 | |  |  | 0.98 |  | | | |  |  | 0.59 | |  |  | -1.12 | |  |  | 2.30 | |  |  | 0.67 | |  |  | 0.50 | |  | | |  | - | 0.05 | |  | - | 1.29 | |  |  | 1.19 |  | - | 0.08 |  |  | 0.93 |  |
| rank [mid] |  |  | 0.60 |  | - | 0.37 | |  |  | 1.56 | |  |  | 1.21 | |  |  | 0.22 |  | | | |  | - | 0.75 | |  | - | 2.35 | |  |  | 0.85 | |  | - | 0.92 | |  |  | 0.36 | |  | | |  |  | 0.64 | |  | - | 0.39 | |  |  | 1.67 |  |  | 1.21 |  |  | 0.23 |  |
| age category [old] |  | - | 0.03 |  | - | 0.83 | |  |  | 0.77 | |  | - | 0.07 | |  |  | 0.95 |  | | | |  |  | ‒ | |  |  | ‒ | |  |  | ‒ | |  |  | ‒ | |  |  | ‒ | |  | | |  | - | 0.13 | |  | - | 1.03 | |  |  | 0.77 |  | - | 0.28 |  |  | 0.78 |  |
| age category [very old] |  | - | 0.74 |  | - | 1.76 | |  |  | 0.27 | |  | - | 1.44 | |  |  | 0.15 |  | | | |  |  | ‒ | |  |  | ‒ | |  |  | ‒ | |  |  | ‒ | |  |  | ‒ | |  | | |  |  | -0.57 | |  |  | -1.69 | |  |  | 0.55 |  |  | -1.00 |  |  | 0.32 |  |
| degree |  | - | 1.44 |  | - | 3.19 | |  |  | 0.30 | |  | - | 1.62 | |  |  | 0.11 |  | | | |  |  | ‒ | |  |  | ‒ | |  |  | ‒ | |  |  | ‒ | |  |  | ‒ | |  | | |  | - | 1.36 | |  | - | 3.30 | |  |  | 0.57 |  | - | 1.38 |  |  | 0.17 |  |
| eigenvector |  |  | **4.09** |  |  | **1.64** | |  |  | **6.53** | |  |  | **3.27** | |  |  | **0.00** |  | | | |  |  | **6.03** | |  |  | **1.67** | |  |  | **10.39** | |  |  | **2.71** | |  |  | **0.01** | |  | | |  |  | **3.63** | |  |  | **1.12** | |  |  | **6.14** |  |  | **2.84** |  |  | **0.00** |  |
| betweenness |  |  | 0.02 |  | - | 2.40 | |  |  | 2.44 | |  |  | 0.02 | |  |  | 0.99 |  | | | |  |  | ‒ | |  |  | ‒ | |  |  | ‒ | |  |  | ‒ | |  |  | ‒ | |  | | |  | - | 0.66 | |  | - | 3.66 | |  |  | 2.33 |  | - | 0.43 |  |  | 0.66 |  |
| closeness |  |  | 0.06 |  | - | 2.64 | |  |  | 2.77 | |  |  | 0.04 | |  |  | 0.96 |  | | | |  |  | 1.64 | |  |  | -5.60 | |  |  | 2.33 | |  |  | -0.81 | |  |  | 0.42 | |  | | |  |  | 1.15 | |  | - | 1.60 | |  |  | 3.90 |  |  | 0.82 |  |  | 0.41 |  |

All models were built using binomial generalized linear mixed models with a log link function and included random effect of Japanese macaque identity (ID). The mixed-network model included social centrality variables calculated within a network that included both males and females. The male-network model derived centrality variables exclusively from male-male social network, and the female-network model did so from female-female social network. Bold values indicate significant effects. For sex, rank, and age category, the reference categories were “female”, “high”, and “adult”, respectively.

References

1. Fushing H, McAssey MP, Beisner B, McCowan B. Ranking network of a captive rhesus macaque society: A sophisticated corporative kingdom. *PloS one* **6**, (2011).

2. Beisner BA, Hannibal DL, Finn KR, Fushing H, McCowan B. Social power, conflict policing, and the role of subordination signals in rhesus macaque society. *American journal of physical anthropology* **160**, 102-112 (2016).

3. Fujii K*, et al.* Perc: Using percolation and conductance to find information flow certainty in a direct network., (2021).

4. Joly M, Micheletta J, De Marco A, Langermans JA, Sterck EHM, Waller BM. Comparing physical and social cognitive skills in macaque species with different degrees of social tolerance. *Proceedings of the Royal Society B-Biological Sciences* **284**, (2017).

5. Herrmann E, Hare B, Call J, Tomasello M. Differences in the cognitive skills of bonobos and chimpanzees. *PloS one* **5**, e12438 (2010).

6. Schmitt V, Pankau B, Fischer J. Old world monkeys compare to apes in the primate cognition test battery. *PloS one* **7**, e32024 (2012).

7. Herrmann E, Call J, Hernandez-Lloreda MV, Hare B, Tomasello M. Humans have evolved specialized skills of social cognition: The cultural intelligence hypothesis. *Science* **317**, 1360-1366 (2007).

8. Diamond A. Executive Functions. *Annual Review of Psychology* **64**, 135-168 (2013).

9. Flombaum JI, Santos LR. Rhesus monkeys attribute perceptions to others. *Current biology : CB* **15**, 447-452 (2005).

10. MacLean EL*, et al.* The evolution of self-control. *Proceedings of the National Academy of Sciences of the United States of America* **111**, E2140-E2148 (2014).
